# The PATH Protocol for Integrated Facial Rejuvenation: A Preliminary Three-Patient Case Report Series and Narrative Review of the Literature

**DOI:** 10.3390/reports9030282

**Published:** 2026-08-25

**Authors:** Enrica Filigheddu, Luigi Sardellitti, Manuela Astrid Chessa, Edoardo Filigheddu, Alessio Pirino, Egle Patrizia Milia

**Affiliations:** 1 Dental Unit, Head and Neck Department, Azienda Ospedaliero Universitaria, 07100 Sassari, Italy; enrica.filigheddu@aouss.it; 2 Kalos Medical Group, 07100 Sassari, Italy; manuelaastridchessa@icloud.com; 3 Plastic Surgery Unit, Department of Medical, Surgical and Experimental Sciences, University Hospital Trust of Sassari, 07100 Sassari, Italy; edofili@gmail.com; 4 Centre for Research and Development in Aesthetic Medicine, Nutraceuticals and Cosmetology, Department of Biomedical Science, University of Sassari, 07100 Sassari, Italy; axelpir@uniss.it; 5 Department of Medicine, Surgery and Pharmacy, University of Sassari, 07100 Sassari, Italy; emilia@uniss.it

**Keywords:** case report, case series, facial rejuvenation, PATH protocol, hyaluronic acid

## Abstract

**Background and Clinical Significance:** Facial aging is a multifactorial process involving the skin, subcutaneous tissues, facial fat compartments, muscles, ligaments, and skeletal structures. Integrated minimally invasive protocols are increasingly used to improve facial harmony and skin quality while preserving natural expression. The PATH (Profundity, Action, Timing, and Home care) protocol combines chemical peeling, hyaluronic acid–succinate filler, intradermal biorevitalization, and post-procedural homecare in a sequential and individualized approach to facial rejuvenation; **Case Presentation:** Three female patients aged 52–58 years with clinical signs of facial aging were treated according to the PATH protocol. Assessments were performed at baseline and after 60 days using standardized photography, OBSERV 520^®^, Antera 3D PRO^®^, and QuantifiCare LifeViz^®^ Infinity Pro. No serious adverse events or systemic complications were reported during the 60-day follow-up period. Mild edema, erythema, and ecchymosis resolved spontaneously within 48–72 h. At 60 days, all patients showed natural improvement in facial appearance, with better midface and lower-face balance, increased skin brightness, improved texture, and no overcorrection or alteration of facial expression. Instrumental evaluations supported the clinical findings, showing improvements in skin regularity, chromatic uniformity, microrelief, and soft-tissue distribution; **Conclusions:** This preliminary case series suggests that the PATH protocol may represent a coherent multimodal strategy for integrated facial rejuvenation. The main clinical lesson is that a sequential, depth-oriented, and individualized approach may achieve natural aesthetic improvement. No serious adverse events were reported in the three patients during the 60-day follow-up period; however, the limited sample size and short follow-up do not allow definitive conclusions regarding safety. Further controlled studies with larger samples and longer follow-up are needed to confirm these exploratory findings.

## 1. Introduction and Clinical Significance

Modern aesthetic medicine has progressively evolved from a purely corrective discipline into an integrated medical approach aimed at preserving tissue function, facial harmony, and psychophysical well-being. Rather than focusing exclusively on the reduction in visible signs of aging, contemporary aesthetic medicine emphasizes prevention, improvement of tissue quality, and modulation of the biological processes involved in cutaneous and facial aging. From this perspective, the face is considered a dynamic anatomical unit composed of the skin, subcutaneous fat, muscles, retaining ligaments, and skeletal structures, whose progressive modifications contribute to the clinical perception of aging [1]. Facial aging is a multifactorial process resulting from the interaction between intrinsic biological mechanisms and extrinsic environmental factors. Intrinsic aging is associated with cellular senescence, hormonal changes, oxidative stress, mitochondrial dysfunction, and reduced fibroblast biosynthetic activity. By contrast, extrinsic aging, particularly photoaging, is mainly driven by ultraviolet radiation, pollution, and smoking, which accelerate oxidative damage, chronic low-grade inflammation, and extracellular matrix degradation [2,3].

At the cutaneous level, aging is characterized by epidermal barrier impairment, reduced cellular turnover, dermal thinning, disorganization of collagen and elastic fibers, reduction in glycosaminoglycan content, and flattening of the dermal–epidermal junction. Dermal fibroblasts play a central role in extracellular matrix homeostasis, and their activity is regulated by biochemical and mechanical signals, including the TGF-β/Smad pathway and integrin-mediated mechanotransduction. With aging and ultraviolet exposure, impaired TGF-β signaling, increased matrix metalloproteinase activity, and altered fibroblast–matrix interactions contribute to progressive matrix degradation and reduced dermal regenerative capacity [4,5,6,7].

Facial aging also involves deeper anatomical structures. Skeletal remodeling, redistribution and atrophy of facial fat compartments, weakening of the retaining ligaments, and changes in muscular tone contribute to volume loss, tissue ptosis, accentuation of facial folds, and loss of mandibular and zygomatic definition. In women, hormonal changes, particularly the decline in estrogen levels during menopause, may further contribute to reduced collagen content, impaired water retention, and loss of skin elasticity [8,9,10,11,12,13,14,15].

In recent years, combined minimally invasive approaches have gained increasing relevance in aesthetic medicine. Chemical peels and cross-linked hyaluronic acid fillers are established procedures supported by a comparatively broad body of clinical evidence and are commonly used to promote epidermal renewal and restore facial volume and contours, respectively. By contrast, the evidence supporting skin boosters, non-cross-linked hyaluronic acid formulations, and products combining hyaluronic and succinic acids is less extensive and more heterogeneous. Although preliminary clinical findings suggest that these approaches may improve skin hydration, firmness, elasticity, and overall skin quality, the specific contribution of succinic acid has not yet been clearly established and requires further investigation [16,17,18].

The PATH protoco was developed by mesoestetic^®^ as a manufacturer-associated therapeutic strategy. The protocol is based on the sequential combination of treatments acting at different anatomical depths, selected according to their intended action and administered according to predefined treatment timings, together with post-procedural home care. It has previously been described and clinically applied, including in the treatment of the periocular region [19].

In the present case series, the PATH framework was individualized according to each patient’s clinical characteristics and pattern of facial aging and included chemical peeling, hyaluronic acid-based filler treatment, intradermal biorevitalization, and post-procedural home care. The rationale underlying this combined approach is that its individual components may complement one another by addressing different clinical features of facial aging, including epidermal appearance, skin hydration, facial volume distribution, and post-procedural skin care. Nevertheless, potential effects on extracellular matrix remodeling, soft-tissue support, and skin-barrier function should be regarded as component-specific biological rationales or theoretical assumptions rather than as established outcomes of the combined PATH protocol.

The present article is a descriptive case series involving three patients. A focused narrative overview of the available literature is included solely to contextualize the biological and clinical rationale of the individual treatments used and is not intended to constitute a standalone narrative review. Accordingly, the primary aim of this article is to describe the clinical application and short-term outcomes of an individualized PATH protocol in three selected cases. Moreover, the novelty of the present report lies not in the development of the PATH concept itself, but in its independent clinical characterization as an individualized, multilayer full-face rejuvenation framework, together with detailed documentation of its practical implementation and complementary clinical, multispectral, quantitative skin, and three-dimensional facial assessments.

### 1.1. Literature Review and Rationale of the PATH Protocol

To contextualize the biological, anatomical, and clinical rationale of the PATH protocol, a focused narrative literature review was conducted using PubMed/MEDLINE, Scopus, and Google Scholar. Publications issued between January 2020, and August 2026 were considered. Older seminal articles were also included when necessary to describe foundational anatomical, biological, rheological, or historical concepts.

The search combined the following keywords using the Boolean operators “AND” and “OR”: “hyaluronic acid filler”, “dermal filler”, “skin booster”, “biorevitalization”, “non-cross-linked hyaluronic acid”, “chemical peeling”, “succinic acid”, “succinate”, “facial rejuvenation”, “full-face approach”, “facial aging”, “skin quality”, “skin aging”, “dermal biostimulation”, “filler rheology”, and “facial anatomy”. Search terms were adapted to the syntax of each database. Reference lists of relevant publications were also manually screened to identify additional eligible articles. Retrieved records were initially screened by title and abstract by two authors (E.F. and L.S.).

Publications considered potentially relevant were subsequently assessed in full text. Articles were selected when they directly addressed at least one of the main topics of the review, including facial aging, hyaluronic acid fillers, filler rheology, chemical peeling, biorevitalization, succinic acid-containing formulations, facial anatomy, treatment safety, or multimodal facial rejuvenation. Priority was given to peer-reviewed clinical studies, systematic and narrative reviews, consensus statements, clinical guidelines, and larger or more recent clinical studies. Relevant preclinical studies were included when clinical evidence was limited and when they provided mechanistic information relevant to the treatment components discussed.

Publications were excluded when they were not directly relevant to the scope of the review, provided insufficient methodological or clinical information, consisted only of conference abstracts, were purely promotional or commercial in nature, or substantially duplicated evidence already represented by more comprehensive publications. Any uncertainty regarding study eligibility was resolved by discussion and consensus between the two authors.

A total of 32 publications were ultimately included in the narrative synthesis. Given the narrative nature of the review, no formal risk-of-bias assessment, evidence grading, or quantitative synthesis was performed. Study selection was performed by two authors, E.F and L.S, and any uncertainty was resolved by consensus between the reviewers.

The findings were synthesized narratively according to the main clinical and biological components of the PATH protocol. Manufacturer-associated sources were used only to document the origin and previous description of the PATH protocol and not to support claims regarding clinical efficacy or safety.

#### 1.1.1. Hyaluronic Acid-Based Treatments and Full-Face Rejuvenation

Within minimally invasive facial rejuvenation, dermal fillers have supported the transition from isolated wrinkle correction toward integrated strategies aimed at restoring facial contours and improving skin quality. The historical evolution of fillers reflects a progressive shift from permanent materials, such as paraffin, silicone, and other synthetic fillers, toward safer and more biocompatible resorbable products because of complications including migration, granulomatous reactions, chronic inflammation, infection, and tissue necrosis [20,21]. Earlier injectable filling materials were associated with a range of inflammatory, granulomatous, and hypersensitivity reactions, contributing to the progressive transition toward newer resorbable materials [22].

The introduction of hyaluronic acid (HA)-based fillers marked a major turning point in aesthetic medicine. HA is a naturally occurring glycosaminoglycan of the extracellular matrix, characterized by biocompatibility, hydrophilicity, low immunogenicity, physiological degradability, and reversibility through hyaluronidase [23]. Both endogenous and exogenous HA are progressively metabolized by endogenous hyaluronidases, cellular turnover, and oxidative processes, while recombinant hyaluronidase allows enzymatic degradation in cases of overcorrection, nodules, irregularities, or vascular complications [24,25,26].

HA used in aesthetic medicine can be broadly divided into linear, non-cross-linked HA and cross-linked HA. Linear HA is mainly used in biorevitalization and skin-quality treatments because of its hydrating and biological properties, including interaction with receptors such as CD44, RHAMM, and TLR2/4, but it has limited tissue persistence because of rapid degradation [24,27,28,29].

Cross-linked HA fillers were developed to prolong tissue persistence and provide structural support. BDDE is currently one of the most widely used cross-linking agents in commercial HA fillers, and the degree of cross-linking influences cohesivity, viscosity, elasticity, duration, and clinical indication. Residual cross-linker-related compounds and degradation by-products are also relevant considerations in the safety assessment of BDDE-cross-linked HA fillers [30,31,32,33].

The clinical behavior of HA fillers is also determined by rheological properties such as G′, G″, cohesivity, hydrophilicity, HA concentration, particle distribution, and tan δ. These parameters guide product selection according to anatomical district, injection depth, tissue mobility, and desired clinical effect [34,35,36]. More structured fillers are generally preferred for deep support and projection, whereas softer and more adaptable gels are preferred for dynamic areas and superficial refinement.

Modern facial rejuvenation increasingly relies on a full-face approach rather than isolated correction of single defects. Facial aging involves skeletal remodeling, fat-compartment redistribution and atrophy, ligamentous laxity, soft-tissue ptosis, and progressive cutaneous aging [12,15,37]. Therefore, isolated correction of lips, nasolabial folds, or the zygomatic region may produce partial or unnatural results if support vectors and facial proportions are not respected. A sequential approach, progressing from deep structural support to superficial refinement, may better restore global harmony while preserving facial tridimensionality and natural expression [38].

#### 1.1.2. Anatomical Safety and Treatment Planning

Anatomical safety remains essential in injectable aesthetic medicine. The facial vascular network is complex and variable, with multiple anastomoses between the external and internal carotid systems. Clinically relevant high-risk areas include the glabella, nasal dorsum, nasolabial and paranasal regions, lips, tear trough, chin, and forehead. Intravascular injection or vascular compression may lead to ischemia, skin necrosis, or, rarely, visual impairment and blindness. Prevention relies on detailed anatomical knowledge, correct injection plane, small aliquots, low-pressure injection, slow retrograde delivery, and early recognition and management of vascular occlusion according to established emergency protocols [26,39,40,41,42].

Sensory innervation of the face is provided by the trigeminal nerve through its ophthalmic, maxillary, and mandibular branches. The infraorbital, supraorbital, supratrochlear, mental, and zygomaticofacial foramina represent relevant anatomical landmarks because neurovascular structures emerge at these sites and may be vulnerable during injections. Although permanent nerve injury is uncommon, knowledge of vascular and neural anatomy is essential to reduce ischemic, neurological, and sensory complications during filler and biostimulatory treatments [39].

#### 1.1.3. HA–Succinate, Chemical Peeling, and Biorevitalization

Succinic acid is an endogenous metabolite involved in mitochondrial metabolism and energy balance. In dermatology and aesthetic medicine, succinate has gained interest because of its potential contribution to tissue repair and dermal homeostasis through mitochondrial metabolism, oxidative pathways, and cellular stress responses. The rationale for combining HA and succinate is based on a potential synergy between the structural effect of HA and the metabolic support provided by succinate. Cross-linked HA contributes to tissue support, hydration, and improvement of skin texture, whereas succinate, as an intermediate of the Krebs cycle, participates in mitochondrial energy production and cellular metabolic regulation [17,43].

In dermal fibroblasts, efficient oxidative metabolism is required for the synthesis of extracellular matrix components, including collagen, elastin, and endogenous HA. Since skin aging and photoaging are associated with mitochondrial dysfunction, oxidative stress, and reduced fibroblast biosynthetic capacity, succinate may help support the metabolic environment required for matrix synthesis, without necessarily acting as a direct pharmacological inducer of mitochondrial biogenesis [43]. Experimental evidence suggests that the association of succinate with HA may be related to increased expression of extracellular matrix genes, including COL1A1 and FBN1, and reduced expression of catabolic mediators such as MMP-1 and pro-inflammatory cytokines associated with the senescent phenotype [17,44].

The effect of succinate on TGF-β/Smad signaling should be interpreted cautiously, as it appears to be indirect and dependent on the cellular metabolic and redox context. In experimental models of fibroblasts exposed to oxidative stress or ultraviolet irradiation, succinate exposure has been associated with increased TGF-β1 expression and Smad2/3 phosphorylation, together with increased anabolic matrix markers and reduced expression of MMP-1, IL-6, and IL-8 [17,45]. HA–CD44 signaling may also contribute to this potential synergy, as CD44 is involved in fibroblast activity, cell proliferation, and modulation of TGF-β-mediated responses [44,46,47]. However, available clinical evidence remains limited, and further controlled studies are needed to clarify the magnitude, duration, and reproducibility of these effects.

Chemical peeling is commonly used in aesthetic dermatology to improve skin texture, brightness, pigmentation irregularities, superficial wrinkles, acne, and photoaging. In combined protocols, peeling may act as a preparatory step before injectable treatments by improving epidermal turnover, skin permeability, and biological responsiveness of the dermo-epidermal unit. Its effect is based on controlled and depth-dependent chemical injury followed by re-epithelialization, fibroblast activation, and extracellular matrix remodeling [48]. Superficial and medium-depth peels are commonly used to improve brightness, dyschromia, texture, fine wrinkles, and superficial scars, while deeper peels are associated with longer recovery and higher risk of adverse events, including systemic toxicity in the case of phenol [48,49].

Biorevitalization is a non-volumizing intradermal technique aimed at improving skin quality through the administration of bioactive substances directly into the dermis. It is intended to support fibroblast activity and endogenous production of collagen, elastin, and glycosaminoglycans, with potential effects on hydration, tone, elasticity, and brightness [50]. Commonly used agents include linear HA, polynucleotides, amino acids, vitamins, biomimetic peptides, coenzymes, minerals, and antioxidants. Polynucleotides may activate purinergic adenosine receptors, particularly A2A receptors, modulating pathways involved in tissue repair, inflammation regulation, and angiogenesis [51,52,53]. However, clinical evidence specifically supporting polynucleotides in aesthetic biorevitalization remains limited and partly extrapolated from preclinical and wound-healing models; therefore, they should be interpreted primarily as biological support for dermal homeostasis rather than as definitive regenerative treatments.

#### 1.1.4. Rationale of the PATH Protocol

The PATH protocolwas developed by mesoestetic^®^ as a manufacturer-associated framework for the sequential planning of combined minimally invasive aesthetic treatments. The protocol has previously been described and clinically applied, including in the treatment of the periocular region [19]. However, its previous use should not be interpreted as formal or independent validation, since the currently available evidence remains limited and predominantly manufacturer associated.

The present study does not introduce the original PATH concept but describes its individualized clinical application in three patients with different patterns of facial aging. The specific products and procedures used in the present cases are reported in the clinical protocol and treatment sections. Any financial support, provision of products, consultancy relationship, or other involvement of the manufacturer is also disclosed in the Funding and Conflicts of Interest statements, as applicable.

Within this framework, Profundity refers to the selection of the intended anatomical treatment plane, ranging from epidermal and dermal interventions to deeper filler placement. Action refers to the intended complementary roles of the individual procedures, including epidermal renewal, improvement of skin hydration and quality, and restoration of facial volume. Time refers to the sequential scheduling of the procedures according to the expected tissue response and the clinical characteristics of the patient. Homecare refers to the post-procedural skin-care phase intended to support recovery and maintain the cosmetic results obtained.

These four dimensions constitute a conceptual framework for treatment planning and should not be interpreted as independently validated mechanisms or established clinical effects of the combined protocol. In the present case series, the PATH framework was adapted to each patient’s anatomical characteristics, degree of facial aging, skin condition, and aesthetic treatment objectives.

## 2. Case Presentation

### 2.1. Case-Series Design, Retrospective Case Identification, and Ethics

This study reports a retrospective, single-center, descriptive case series based on routinely collected clinical data from three female patients who underwent facial rejuvenation treatment according to the PATH framework at Kalos Medical Group, Sassari between 16 October 2025 and 15 December 2025.

The patients were treated exclusively as part of routine outpatient clinical care and were not recruited for a research study. The decision to review and publish these cases was made only after the treatments and the available routine follow-up assessments had been completed. No treatment procedure, treatment interval, follow-up visit, photographic acquisition, instrumental examination, or patient-reported assessment was prescribed or scheduled specifically for research purposes.

Treatment decisions were made before and independently of the decision to publish the cases and were based on each patient’s clinical characteristics, facial aging pattern, skin condition, aesthetic concerns, and preferences. Clinical records, standardized photographs, instrumental examinations, and patient-reported information collected during routine clinical practice were subsequently reviewed retrospectively.

The objective of the present report was to describe the individualized clinical application and short-term outcomes of the PATH framework in three routine-care cases. Changes in facial appearance, facial volume distribution, and skin characteristics were assessed using the available clinical, photographic, and instrumental documentation obtained at baseline and at the routine final follow-up visit.

Treatment-related local and systemic events documented during the available follow-up period were also retrospectively collected and reported descriptively. Given the inclusion of only three patients and the limited follow-up period, this case series was not designed to assess or establish the safety profile of the PATH protocol.

All patients provided written informed consent for the clinical procedures and routine photographic documentation before treatment. Following the subsequent decision to publish the cases, separate written informed consent was obtained from each patient for publication of clinical information and potentially identifiable full-face photographs. Patients were informed that masking the eye region could not guarantee complete anonymity.

No inferential statistical analysis was performed because of the retrospective descriptive design and the inclusion of only three cases.

### 2.2. Treatment Products, Composition, and Supply

The quantitative composition of the injectable products and the complete International Nomenclature of Cosmetic Ingredients, INCI, composition of the topical products used in the three patients are reported in Table 1. The information was obtained from the manufacturers’ instructions for use, technical documentation, and product labels.

All products were purchased through the clinic’s routine procurement process and were not supplied by the manufacturer.

Mesoestetic Pharma Group had no role in the clinical decision to treat the patients, treatment planning, retrospective case selection, clinical documentation, data interpretation, manuscript preparation, or the decision to publish.

### 2.3. Patient Characteristics, Clinical Suitability, and Retrospective Case Selection

Three female patients aged 52–58 years were retrospectively identified from the outpatient clinical records. All patients were in the peri- or post-menopausal period and presented with moderate signs of facial aging, including visible changes in skin quality, mild-to-moderate volume loss involving the midface and/or lower face, and mild-to-moderate tissue laxity. None of the patients presented with advanced facial aging or severe tissue ptosis.

Treatment areas were selected according to individual clinical needs and included the zygomatic region, nasolabial folds, marionette lines, mandibular contour, and, when clinically indicated, additional areas as part of an individualized facial harmonization approach. All patients requested improvement in facial appearance while preserving natural expression.

The clinical decision to perform the treatments was made before and independently of the decision to publish the cases. At the time of treatment, patients were considered clinically suitable on the basis of good general health, absence of active inflammatory or infectious skin disease, absence of permanent facial fillers, absence of volumetric facial treatments during the previous 12 months, and absence of known hypersensitivity to the products used.

Clinical contraindications to treatment included pregnancy or breastfeeding, active autoimmune or connective-tissue disease, coagulation disorders, active bacterial or viral infection in the treatment area, active herpes simplex infection, folliculitis, inflammatory acne, and known hypersensitivity to hyaluronic acid, lidocaine, succinate, or any other component of the products used.

All three patients were non-smokers, were not taking anticoagulant or antiplatelet medications, and had not undergone medical or surgical facial aesthetic procedures during the three months preceding treatment.

For the purposes of the present retrospective publication, cases were considered when sufficiently complete and comparable documentation was available, including baseline clinical records, information on the products and volumes administered, standardized photographs, instrumental assessments, and a routine final follow-up assessment at approximately 60 days. These criteria were applied retrospectively and were not used to recruit patients into a research study.

The three patients represented three cases retrospectively selected from a larger group of patients treated during routine clinical practice because sufficiently complete and comparable documentation was available.

Because the cases were retrospectively identified after completion of treatment, the possibility of selection bias cannot be excluded.

### 2.4. PATH Framework and Treatment Procedures

The PATH protocolwas developed by mesoestetic^®^ as a manufacturer-associated framework for the individualized planning of combined minimally invasive aesthetic procedures.

Profundity refers to the selection of the intended anatomical treatment plane. Chemical peeling primarily targets the epidermal surface, intradermal biorevitalization is performed within the dermal compartment, and cross-linked hyaluronic acid filler is placed in deeper subcutaneous or supraperiosteal planes according to the anatomical area and clinical objective.

Action refers to the intended complementary functions of the individual procedures, including epidermal renewal through chemical peeling, non-volumizing intradermal treatment intended to improve skin hydration and appearance, and structural volume restoration using cross-linked hyaluronic acid filler associated with succinic acid.

Time refers to the sequential organization of the procedures according to the individual clinical response, skin tolerance, treatment requirements, and routine appointment availability. Treatment intervals were not established for research purposes. The exact treatment schedule for each patient was retrospectively reconstructed from the clinical records and is reported in Table 2.

Homecare refers to the post-procedural topical regimen prescribed to support recovery, reduce skin irritation, provide photoprotection, and maintain the cosmetic results obtained.

The PATH framework has previously been described and used clinically but has not been independently validated through large, controlled studies. The present case series describes its individualized application in three routine-care patients and should not be interpreted as a formal validation of the protocol.

#### 2.4.1. Chemical Peeling

Mesopeel^®^ MD Azelan RX, mesoestetic^®^, Barcelona, Spain, was used as a superficial chemical peel. The formulation contains azelaic acid (18%), salicylic acid (15%), and lactic acid (5%), together with retinal, glycyrrhetinic acid, tranexamic acid, shikimic acid, caffeic acid, and arginine.

Before application, the facial skin was cleansed using ALLSKIN|MED Purifying Cleansing Gel and rinsed thoroughly with water. The skin was subsequently degreased using three sprays of INNOAESTHETICS^®^ (Laboratorio Innoaesthetics, S.L.U., Sant Just Desvern, Barcelona, Spain) Degreasing Solution applied with cotton pads over the entire face.

The peel was applied in two consecutive layers and maintained for an overall exposure time of 5 min. It was subsequently neutralized using 4–5 sprays of Mesoestetic^®^ Post-Peel Neutralizing Spray, applied with cotton pads over the treated facial areas, followed by removal of any residual product with a water-moistened facial sponge.

Each patient underwent chemical-peeling sessions. The exact treatment dates, number of sessions, and any patient-specific adjustments are reported in Table 2.

#### 2.4.2. HA–Succinate Filler

Mesofiller^®^ Nexha Volume, Mesoestetic Pharma Group, Barcelona, Spain, was used for structural volume restoration. The product contains cross-linked hyaluronic acid at a concentration of 25 mg/mL, succinic acid and lidocaine 0.3%.

According to the manufacturer, the declared rheological parameters include G′ 428 Pa, G″ 91 Pa, and a swelling factor of 1.95 mL/g. Each package contains one 1 mL syringe and one 27 G × 13 mm needle.

Injections were performed using either the supplied 27 G × 13 mm needle or a 25 G × 50 mm blunt cannula, depending on the anatomical region, intended treatment plane, and injection technique.

The filler was administered using linear retrograde threading, fanning, and bolus techniques according to the anatomical area treated. The zygomatic region was treated with supraperiosteal bolus injections using a needle, combined with linear retrograde threading using a cannula. The preauricular region was treated with linear retrograde threading using a cannula in the subcutaneous plane. At the mandibular angle, supraperiosteal boluses were delivered using a needle. The jawline was treated with linear retrograde threading using a cannula in the subcutaneous plane, with additional product deposition toward the chin. The marionette lines and nasolabial folds were treated using linear retrograde fanning with a cannula in the subcutaneous plane. Chin projection was achieved using supraperiosteal bolus injections with a needle.

The anatomical areas treated, injection devices, injection planes, techniques, number of syringes, and exact volumes administered to each patient are reported in Table 2. Mesofiller^®^ Nexha Volume was not mixed with any other injectable product.

#### 2.4.3. Intradermal Biorevitalization

Intradermal biorevitalization was performed using mesohyal™ Organic Silicon and mesohyal™ X-DNA, Mesoestetic Pharma Group, Barcelona, Spain.

Mesohyal™ Organic Silicon is supplied in a 5 mL vial and contains free hyaluronic acid at a concentration of 2.5 mg/mL together with organic silicon (monomethylsilanetriol; organic silicon, 2.7 mg/5 mL).

Mesohyal™ X-DNA is supplied in a 3 mL vial and contains free hyaluronic acid at a concentration of 2.5 mg/mL together with sodium deoxyribonucleotide-derived polynucleotides (PDRN/polynucleotides; concentration not disclosed by the manufacturer).

The products were administered intradermally through zygomatic, mandibular, and subcommissural access points using a 25 G × 50 mm blunt cannula. The exact volume of each product administered during each session and to each patient is reported in Table 2.

Mesohyal™ Organic Silicon and mesohyal™ X-DNA were combined in a single sterile syringe immediately before administration.

The prepared mixture contained 2.5 mL of mesohyal™ Organic Silicon and 3 mL of mesohyal™ X-DNA, for a total prepared volume of 5.5 mL.

The admixture of the two products was consistent with the manufacturer’s written instructions.

#### 2.4.4. Post-Procedural and Homecare Regimen

Immediately after each treatment session, Anti-Stress Mask, mesoestetic^®^, was applied in the clinical setting in a uniform layer covering the entire treated facial area for 10–15 min and subsequently removed using a damp sponge. During the early post-procedural period, patients applied Post-Procedure Gel Cream in an amount corresponding to approximately 1–2 mL per application, twice daily (morning and evening), until complete resolution of erythema and skin reactivity.

After resolution of post-procedural irritation, Age Element Firming Concentrate was introduced on post-treatment day once skin reactivity had completely resolved. Patients applied 3–4 drops twice daily (morning and evening) throughout the treatment period.

Skinretin 0.3% was introduced 5 days after each peeling session, provided that erythema and irritation had completely resolved. A thin layer was applied every other evening and discontinued 5 days before the subsequent peeling session.

Mesoprotech^®^ Water Veil SPF 50+ was applied every morning throughout the treatment period in an amount corresponding to approximately 2 mg/cm^2^ of exposed facial skin. Patients were instructed to reapply the sunscreen every 2 h during prolonged outdoor exposure, as well as after swimming, excessive sweating, or towel drying during prolonged outdoor exposure.

Patients were also advised to avoid direct sun exposure, intense physical activity, saunas, facial massage, and potentially irritating topical products for direct sun exposure throughout the treatment period; intense physical activity, saunas, and facial massage for 48 h after each procedure; and potentially irritating topical products for 5 days after treatment or until complete resolution of erythema and skin irritation, whichever occurred later after each procedure.

Any patient-specific variation in the homecare regimen is reported in Table 2.

### 2.5. Patient-Specific Treatment Schedule and Follow-Up

All treatment dates, procedures, administered products, anatomical areas, injection planes, doses, volumes, and follow-up assessments were retrospectively extracted from the clinical records.

The treatment schedule was individualized according to each patient’s clinical presentation, skin tolerance, response to the previous session, and routine appointment availability. The intervals were not predefined for research purposes.

Further follow-up may have been recommended as part of routine clinical care, but only the documentation available within the observation period analyzed in the present case series was included.

### 2.6. Clinical, Photographic, Patient-Reported, and Instrumental Assessments

Clinical, photographic, patient-reported, and instrumental information collected during routine clinical care was retrospectively reviewed.

Standardized facial photographs were obtained at baseline and at final follow-up using consistent lighting, camera distance, framing, exposure settings, facial expression, and patient positioning.

Clinical and photographic evaluations were performed by the treating physician.

Clinical evaluation considered facial symmetry, facial volume distribution, persistence of correction, overall facial harmony, brightness, texture, and visible skin uniformity. No validated scale was used specifically to assess facial harmony or overall skin quality. These assessments were therefore subjective and descriptive.

#### 2.6.1. Global Aesthetic Improvement Scale

Overall aesthetic improvement compared with baseline was assessed at the final routine follow-up using the five-point Global Aesthetic Improvement Scale, GAIS [54].

The GAIS was completed by the patients and assessed overall perceived aesthetic change according to the following categories:

5 = very much improved;
4 = much improved;
3 = improved;
2 = no change;
1 = worse.

When completed by both the patient and physician, the two evaluations were recorded and reported separately. The GAIS assessed overall aesthetic change and was not used as a specific measurement of facial harmony, skin quality, or treatment safety.

#### 2.6.2. OBSERV 520^®^

OBSERV 520^®^, Eurus, The Netherlands, was used for standardized multispectral photographic acquisition. Available acquisition modes included Daylight, Cross-Polarized, Parallel-Polarized, UV/Wood’s light, True UV, Pigmentation/Brown Spots, Vascularity/Red Areas, and Texture Mode.

In the present case series, OBSERV 520^®^ images were reviewed exclusively for descriptive and iconographic comparison between baseline and final follow-up as part of routine clinical documentation. No quantitative outcome values derived from this device were used.

#### 2.6.3. Antera 3D PRO^®^

Antera 3D PRO^®^, Miravex, Ireland, was used for quantitative assessment of morphological and chromatic skin parameters, including roughness, fine-line depth, texture, furrows, pores, melanin distribution, and hemoglobin-related redness [55,56].

The same facial regions of interest were compared at baseline and final follow-up using identical acquisition settings, patient positioning, and software-defined regions of interest. Percentage variations were generated directly by the device software.

The resulting values were reported descriptively for each patient. No inferential statistical analysis was performed.

#### 2.6.4. QuantifiCare LifeViz^®^ Infinity Pro

QuantifiCare LifeViz^®^ Infinity Pro, QuantifiCare S.A., Biot, France, was used for standardized three-dimensional photographic acquisition and assessment of facial soft-tissue morphology and volume distribution [57,58].

The device-generated parameters included volumetric variation, surface displacement, contour changes, projection, or symmetry.

Quantitative measurements were reported only when directly generated by the software. When numerical outputs were not available, the three-dimensional images were interpreted descriptively.

### 2.7. Treatment-Related Events and Data Analysis

Treatment-related local and systemic events documented in the clinical records during the observation period were retrospectively collected.

Recorded local reactions included edema, erythema, ecchymosis, pain, nodules, and signs of infection. The clinical records were also reviewed for delayed inflammatory reactions, granulomatous reactions, cutaneous necrosis, suspected vascular complications, and systemic events.

When available, the records also included product lot numbers, anatomical areas treated, injected volumes, injection devices, techniques, and subsequent clinical evolution.

These findings were reported only as treatment-related events observed or not observed in the three patients during the available follow-up period. They were not used to assess or establish the safety profile of the PATH protocol.

Clinical, photographic, instrumental, and patient-reported outcomes were analyzed descriptively. Antera 3D PRO^®^ values were reported as percentage variations between baseline and final follow-up. QuantifiCare LifeViz^®^ Infinity Pro results were reported using the quantitative outputs generated by the software and, when numerical data were unavailable, through descriptive three-dimensional comparison. OBSERV 520^®^ images were used exclusively for descriptive and iconographic purposes.

No inferential statistical analysis was performed.

### 2.8. Clinical Case Descriptions

Three female patients aged between were treated according to the PATH protocol. All patients underwent a combined sequential treatment including chemical peeling, HA–succinate filler injection, intradermal biorevitalization, and post-procedural homecare. Clinical, photographic, and instrumental assessments were performed at baseline and at T60, approximately 60 days after the first combined treatment session.

To preserve readability and avoid excessive iconographic redundancy, only representative clinical and instrumental images are included in the main manuscript. Complete standardized photographic documentation and full OBSERV 520^®^, Antera 3D PRO^®^, and QuantifiCare LifeViz^®^ Infinity Pro image panels are provided as Supplementary Figures.

A summary of the main clinical and treatment characteristics of the three cases is reported in Table 3.

#### 2.8.1. Case 1

The first patient was a 52-year-old woman presenting with moderate facial aging involving both cutaneous and structural components. The clinical phenotype was compatible with Glogau photoaging grade II–III, characterized by superficial dyschromia, irregular skin texture, and early static wrinkles. Structurally, the patient showed initial loss of projection in the middle third of the face, mild laxity of the lower third, and reduced definition of anatomical transitions, without severe lipoatrophy or advanced tissue ptosis.

Treatment was planned as a global and progressive approach involving the middle and lower thirds of the face. The patient underwent the combined PATH protocol, with injection of 2 mL of HA–succinate filler. At T60, standardized photographs showed improved facial profile harmony, better continuity of midface volume, increased skin brightness and uniformity, and no evidence of overcorrection or alteration of facial expression.

**Figure 1:**
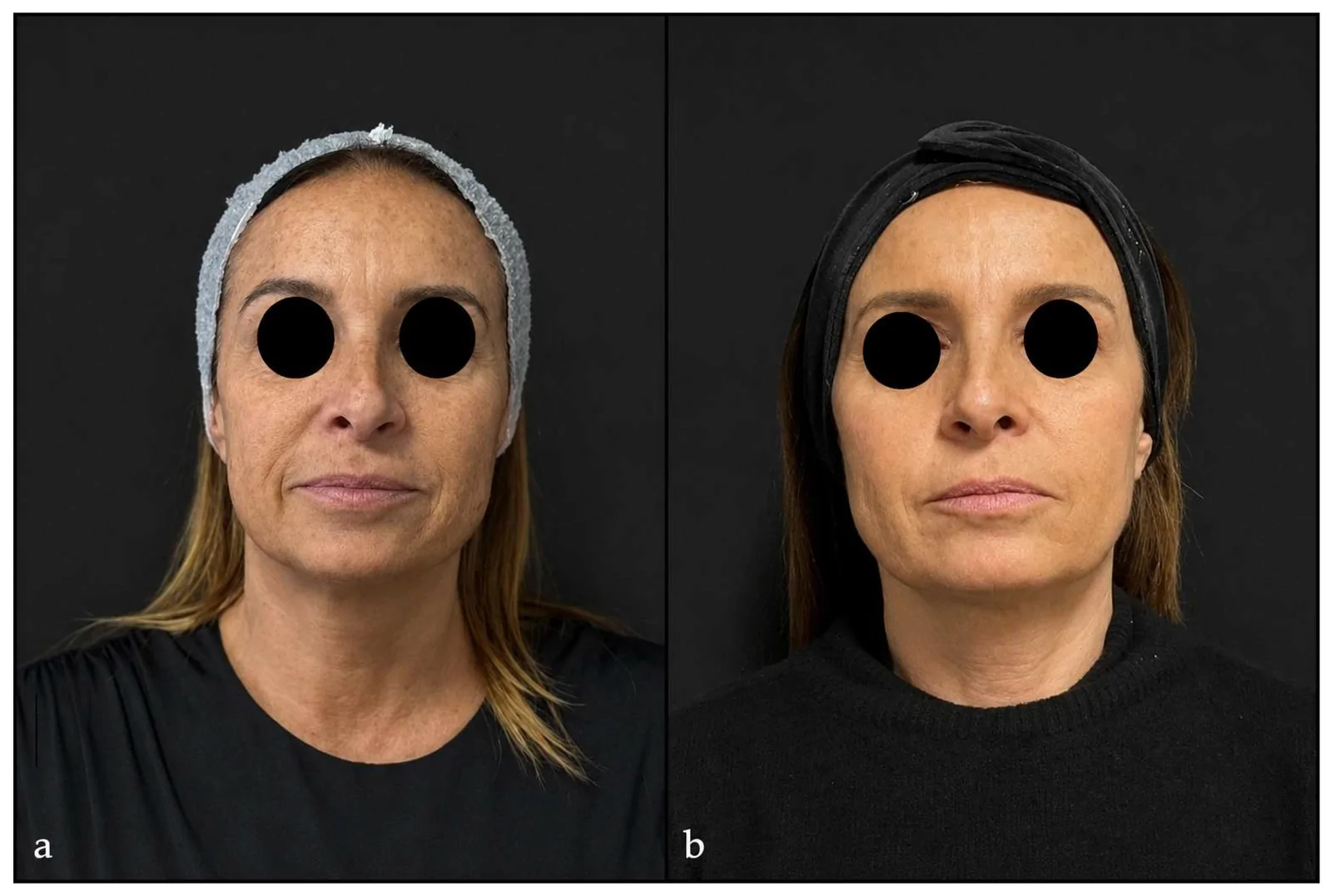
Standardized frontal photographs before treatment (**a**) and at T60 after the PATH protocol (**b**). The images show improved facial harmony, greater continuity of the midface volume, increased skin brightness and uniformity, and no evidence of overcorrection or alteration of facial expression.

**Figure 2:**
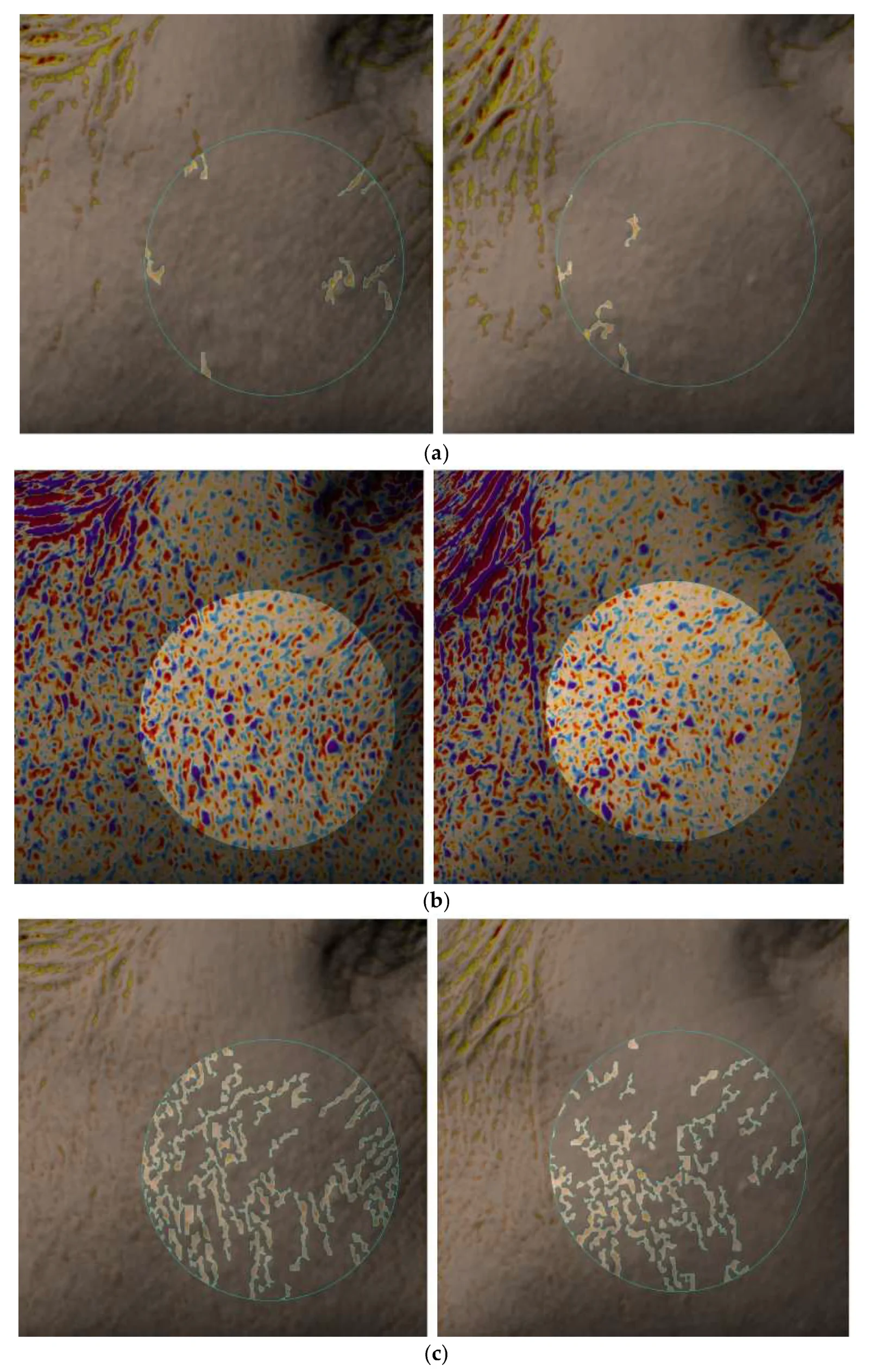
Representative Antera 3D PRO^®^ assessment before treatment and at T60. Selected maps show changes in wrinkles (**a**), skin texture/roughness (**b**), and fine lines/3D surface mapping (**c**). Pre-treatment images are shown in the left column and post-treatment T60 images in the right column. The findings support an improvement in skin surface regularity, microrelief, and overall cutaneous texture after the PATH protocol.

**Figure 3:**
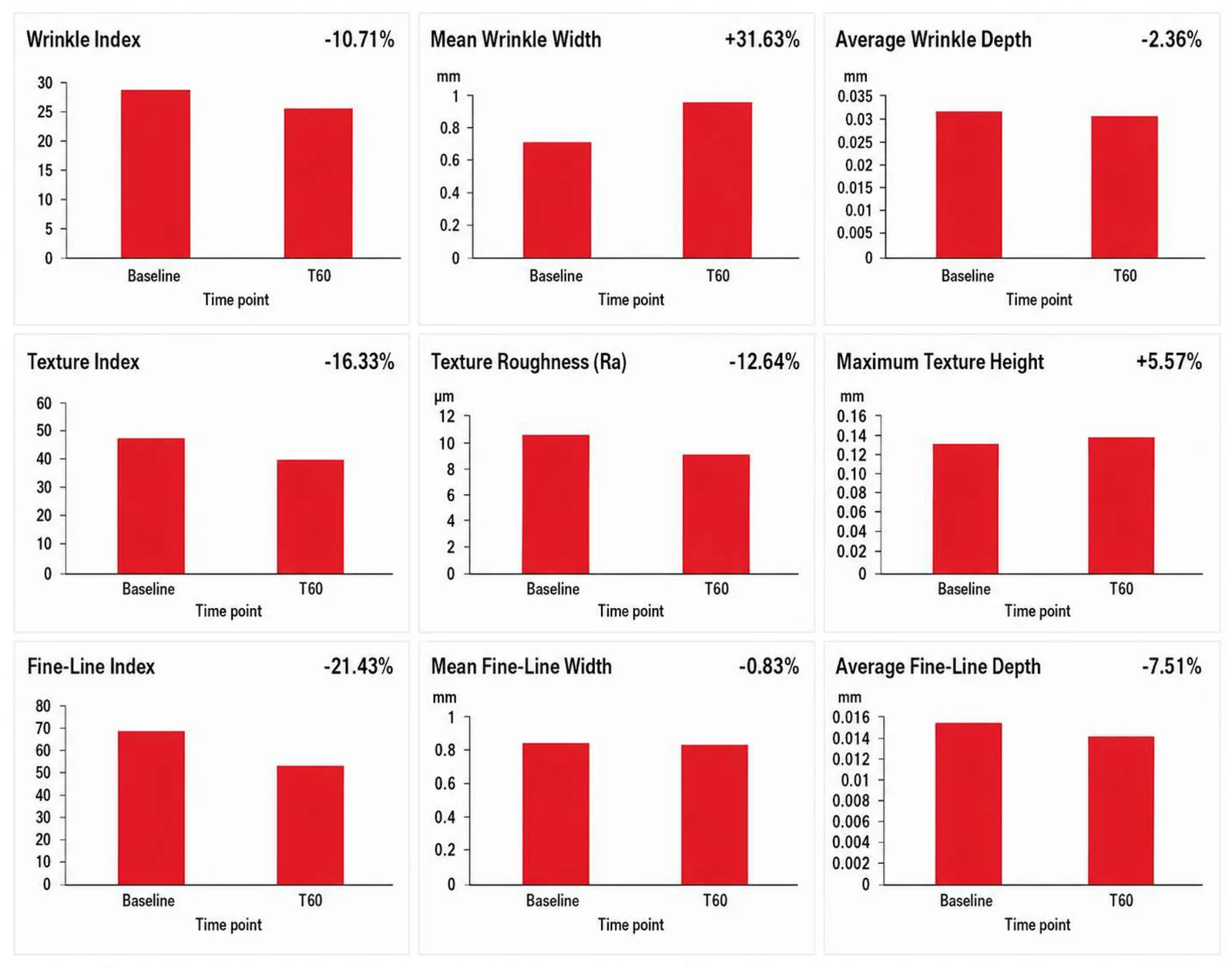
Quantitative Antera 3D PRO^®^ analysis of selected skin-surface parameters between baseline and T60 (60-day follow-up). The charts show descriptive changes in wrinkle index, mean wrinkle width, average wrinkle depth, texture index, texture roughness (Ra), maximum texture height, fine-line index, mean fine-line width, and average fine-line depth. Values were generated directly by the device software and are presented for descriptive comparison between baseline and T60.

Representative clinical and instrumental findings are shown in Figure 1, Figure 2 and Figure 3. Complete standardized photographic documentation and full instrumental image panels are provided in Supplementary Figures.

#### 2.8.2. Case 2

The second patient was a 52-year-old woman presenting with moderate facial aging, mainly involving the perioral and genian regions. The clinical phenotype was compatible with Glogau photoaging grade II–III, with dyschromia, irregular skin texture, and static perioral wrinkles. Structurally, superficial soft-tissue depletion was observed in the genian and perioral regions, without significant deep midface volume deficit, zygomatic projection loss, or relevant mandibular-angle alteration.

Treatment was planned as a selective approach focused on perioral support and improvement of skin quality in the genian region. The patient underwent the combined PATH protocol, with injection of 2 mL of HA–succinate filler. At T60, standardized photographs showed improved perioral volume continuity, reduced wrinkle visibility, and greater chromatic uniformity, without excessive modification of zygomatic projection or mandibular profile.

**Figure 4:**
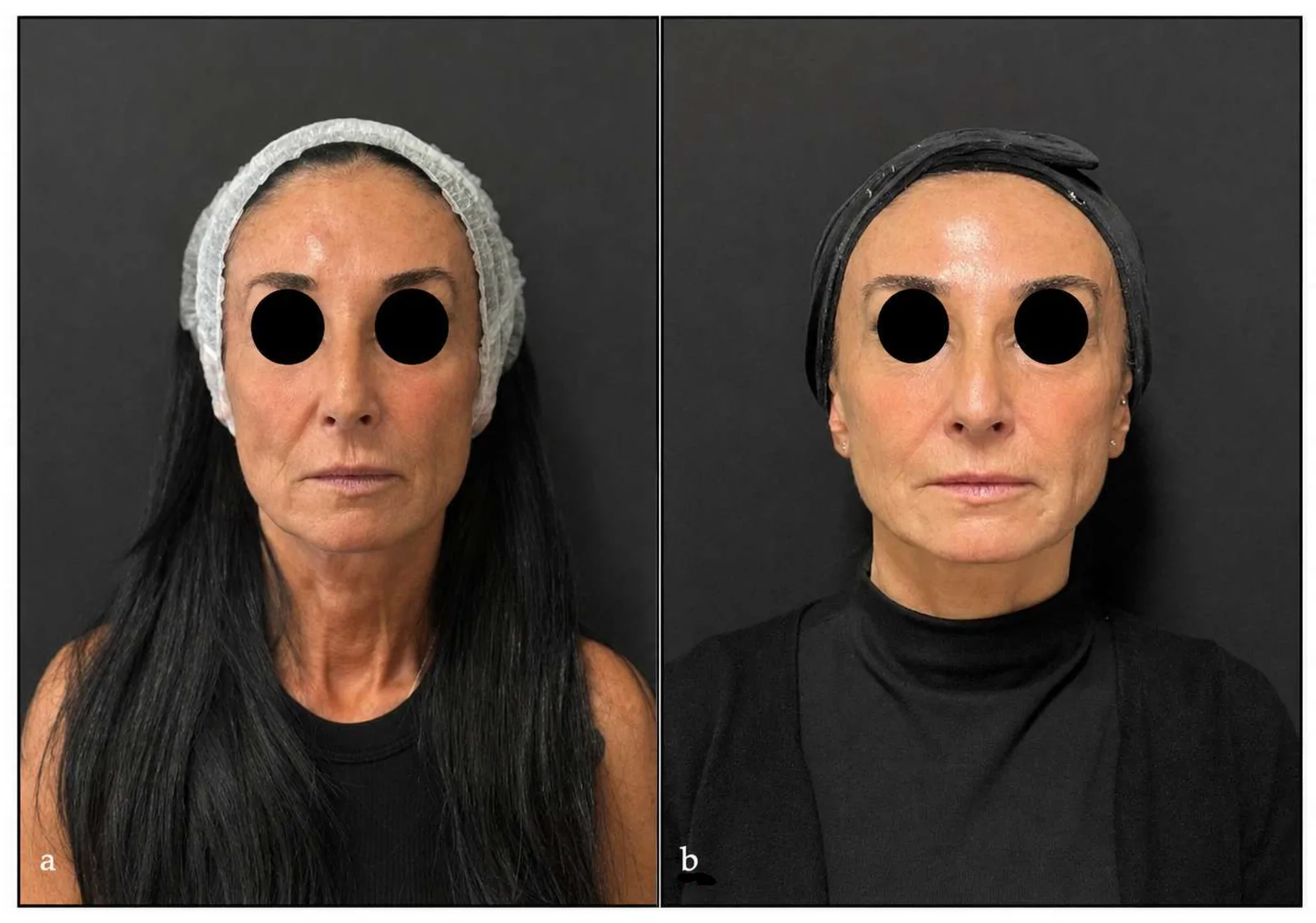
Case 2. Standardized frontal photographs before treatment (**a**) and at T60 after the PATH protocol (**b**), showing improved perioral and genian volume continuity, greater chromatic uniformity, and preservation of natural facial expression without evidence of overcorrection.

**Figure 5:**
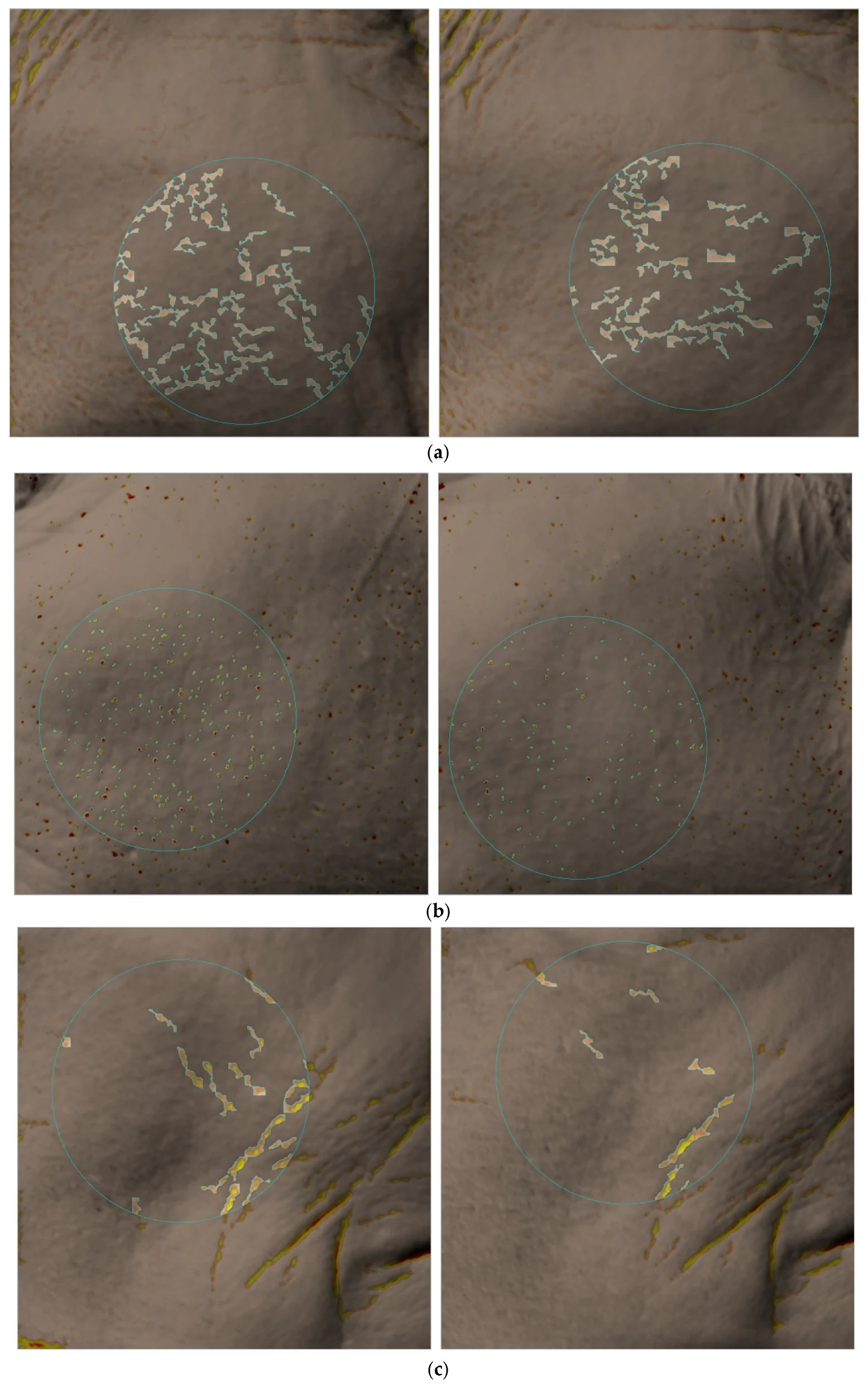
Representative Antera 3D PRO^®^ assessment before treatment and at T60. Selected maps show changes in fine lines (**a**), pores (**b**), and wrinkles (**c**). Pre-treatment images are shown in the left column and post-treatment T60 images in the right column. The findings support improved skin surface regularity, smoother cutaneous texture, and better microrelief in the treated areas after the PATH protocol.

**Figure 6:**
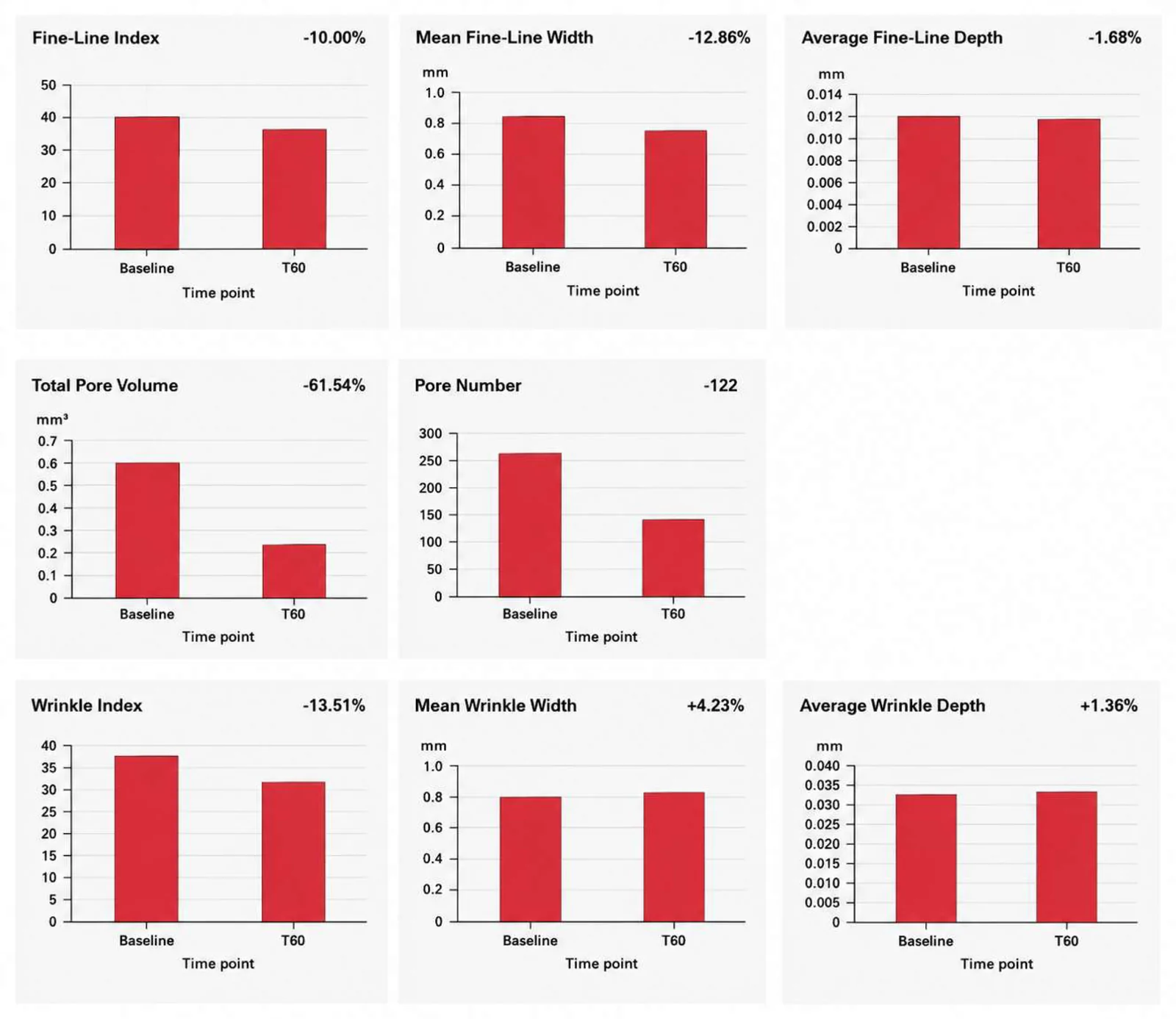
Quantitative Antera 3D PRO^®^ analysis of additional selected skin-surface parameters between baseline and T60 (60-day follow-up). The charts show descriptive changes in fine-line index, mean fine-line width, average fine-line depth, total pore volume, pore number, wrinkle index, mean wrinkle width, and average wrinkle depth. Values were generated directly by the device software and are presented for descriptive comparison between baseline and T60.

Representative clinical and instrumental findings are shown in Figure 4, Figure 5 and Figure 6. Complete standardized photographic documentation and full instrumental image panels are provided in Supplementary Figures.

#### 2.8.3. Case 3

The third patient was a 58-year-old woman presenting with moderate-to-advanced facial aging and predominant structural involvement. The clinical phenotype was compatible with Glogau photoaging grade II–III. Marked laxity of the lower third, reduced midface volume, and loss of definition of anatomical transitions were observed. Compared with the previous cases, photodamage was less evident, with fewer superficial dyschromia and relatively more homogeneous skin tone.

Treatment was planned as a more global and intensive approach involving the middle and lower thirds of the face. The patient underwent the combined PATH protocol, with injection of 4 mL of HA–succinate filler. At T60, standardized photographs showed improved volumetric balance, greater continuity of anatomical transitions, and a more harmonious and natural appearance without signs of overcorrection.

**Figure 7:**
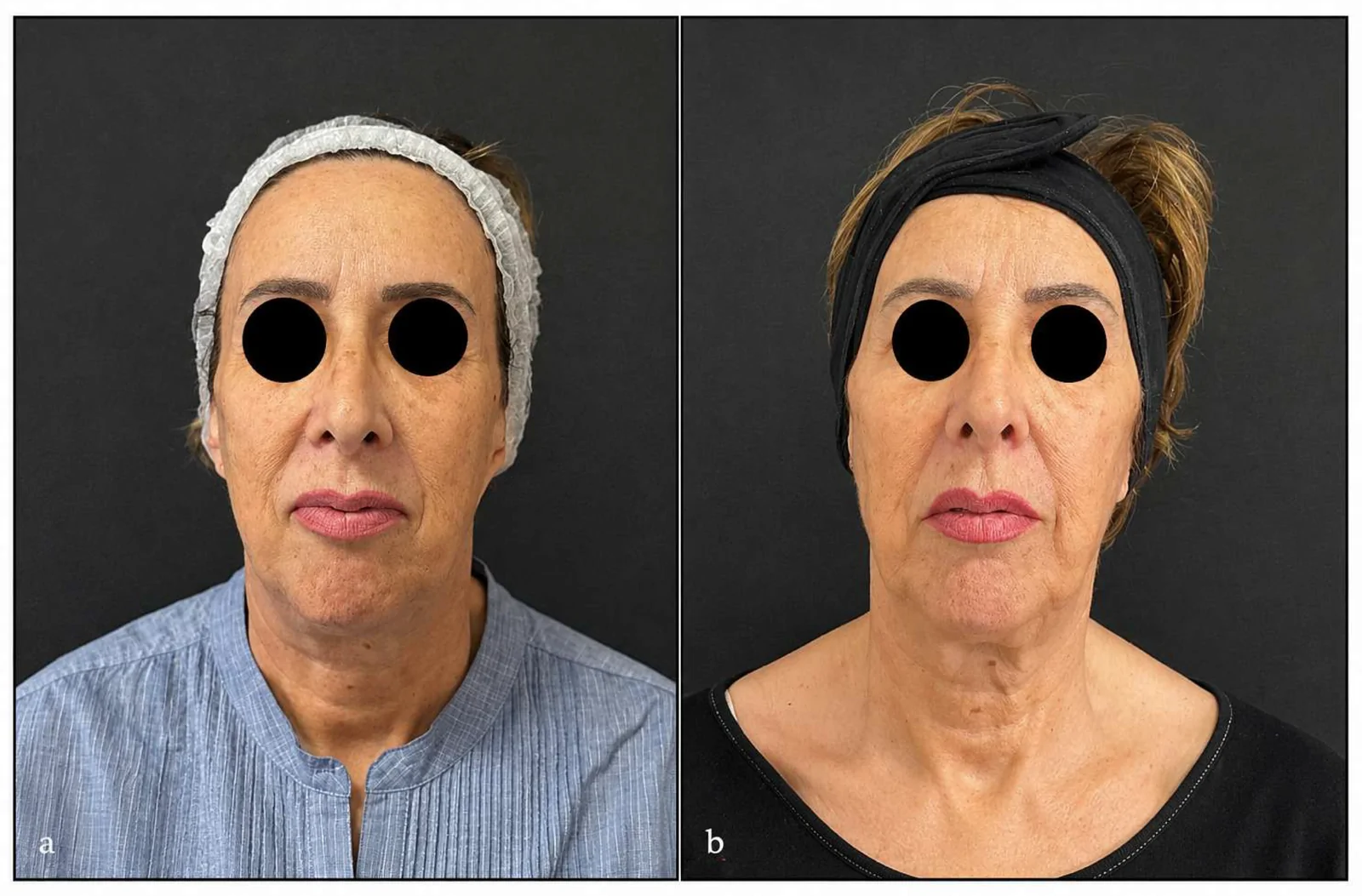
Standardized frontal photographs before treatment (**a**) and at T60 after the PATH protocol (**b**), showing improved volumetric balance, greater continuity of facial contours, and a more harmonious appearance without evidence of overcorrection.

**Figure 8:**
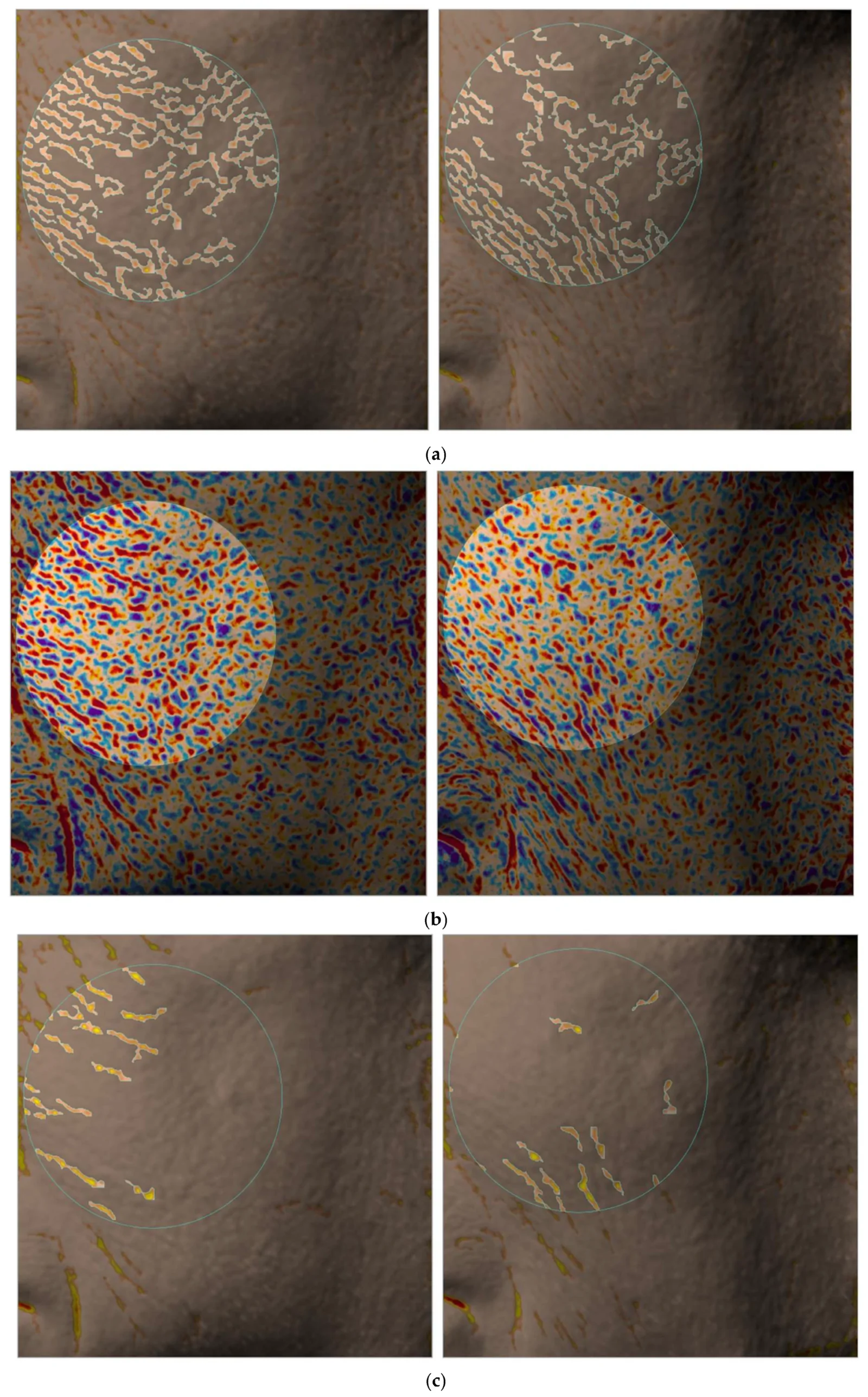
Representative Antera 3D PRO^®^ assessment before treatment and at T60. Selected maps show changes in fine lines/furrows (**a**), skin texture/roughness (**b**), and wrinkles (**c**). Pre-treatment images are shown in the left column and post-treatment T60 images in the right column. The findings support improved skin surface regularity, smoother cutaneous texture, and greater microrelief uniformity after the PATH protocol.

**Figure 9:**
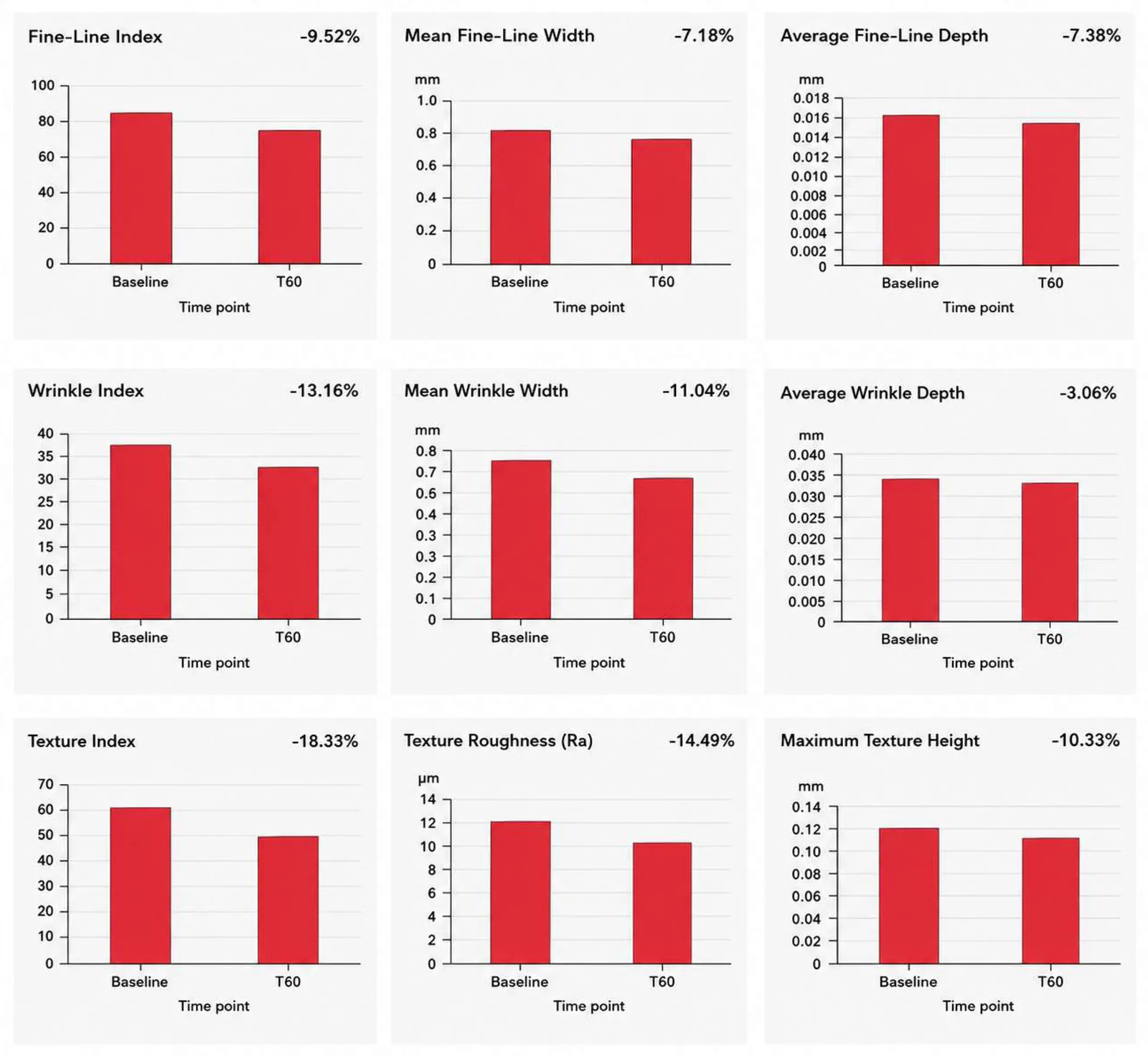
Quantitative Antera 3D PRO^®^ analysis of selected skin-surface parameters between baseline and T60 (60-day follow-up). The charts show descriptive changes in fine-line index, mean fine-line width, average fine-line depth, wrinkle index, mean wrinkle width, average wrinkle depth, texture index, texture roughness (Ra), and maximum texture height. Values were generated directly by the device software and are presented for descriptive comparison between baseline and T60.

Representative clinical and instrumental findings are shown in Figure 7, Figure 8 and Figure 9. Complete standardized photographic documentation and full instrumental image panels are provided in Supplementary Figures.

### 2.9. Clinical Outcomes and Instrumental Assessments

#### 2.9.1. Safety and Tolerability

No clinically significant adverse events or systemic complications were observed in any of the three patients treated with the PATH protocol. Local post-procedural reactions were mild and transient, including edema, erythema, and mild ecchymosis at the injection sites. All reactions resolved spontaneously within 48–72 h, without additional medical intervention.

No nodules, granulomas, infections, cutaneous necrosis, vascular complications, or delayed inflammatory reactions were reported during the 60-day follow-up. Overall, the protocol showed a favorable tolerability profile in this preliminary case series (Table 4).

#### 2.9.2. Clinical and Photographic Outcomes

At T60, standardized photographic documentation showed progressive and harmonious improvement in facial appearance in all three cases. Clinically, the main changes included improved balance of the middle and lower thirds of the face, enhanced malar projection where indicated, attenuation of nasolabial and labiomental folds, and improved mandibular contour definition in patients with greater structural laxity.

The final outcomes appeared natural and consistent with each patient’s anatomical characteristics. No overcorrection, excessive volumization, facial disharmony, or alteration of facial expression was observed (Table 5).

#### 2.9.3. Instrumental Outcomes

Instrumental assessments performed at T60 were consistent with the clinical and photographic findings. OBSERV 520^®^ was used for descriptive and iconographic purposes only, whereas Antera 3D PRO^®^ and QuantifiCare LifeViz^®^ Infinity Pro provided the main quantitative and three-dimensional evaluations.

OBSERV 520^®^ showed qualitative improvement in skin appearance in all cases, with greater chromatic uniformity, improved texture, and reduction in superficial irregularities. In Case 1, the most evident changes involved improved pigmentation uniformity and smoother texture. In Case 2, improvements were mainly observed in the perioral and genian regions, with more homogeneous pigmentation and smoother surface appearance. In Case 3, OBSERV 520^®^ showed improved global texture and chromatic stability during follow-up.

Antera 3D PRO^®^ analysis showed improvement in skin surface quality and microrelief regularity in all three cases. Case 1 showed reduction in fine lines, more regular texture, and improved skin-tone uniformity. Case 2 showed improvement in fine lines, texture, wrinkles, pores, and pigmentation, with a homogeneous response across the analyzed areas. Case 3 showed improvement mainly in malar wrinkles, fine lines, texture, and surface continuity. In this case, increased pigmentation and redness signals were observed in some areas; however, in the absence of clinical dyschromia or persistent inflammation, these changes were interpreted as reactive modulation rather than pathological worsening (Table 6).

QuantifiCare LifeViz^®^ Infinity Pro analysis provided additional information on superficial tissue displacement and volumetric changes. In all cases, vector mapping showed mild and harmonious cranio-lateral tissue displacement, mainly in the midface region, without focal irregularities or clinically relevant asymmetries.

In Case 1, QuantifiCare LifeViz^®^ Infinity Pro was used mainly for descriptive and qualitative purposes because measurable volumetric parameters were not available. In Case 2, indicative volumetric increases of approximately 1.9–2.9 mm were observed in the malar and mandibular regions. In Case 3, bilateral increases of approximately 2.02 mm and 1.87 mm were observed in the zygomatic-malar regions (Table 7).

#### 2.9.4. Patient-Reported Outcomes

Patient-reported outcomes showed a moderate-to-marked perceived improvement after treatment. The GAIS score was recorded as 4/5, corresponding to “much improved” according to the scale adopted in this study. Patients reported skin that appeared more compact, brighter, and more elastic, together with improved aesthetic self-perception and preservation of natural facial expression.

#### 2.9.5. Overall Interpretation of Results

Overall, the PATH protocol was associated with clinical, photographic, and instrumental improvements involving volumetric, structural, and skin-quality parameters, with a favorable safety profile during the 60-day follow-up. The integration of clinical, photographic, instrumental, and patient-reported assessments allowed a multidimensional interpretation of treatment outcomes.

In the present case series, the Glogau scale was used only as a descriptive tool to characterize the degree of cutaneous photoaging. The analyzed cases showed that the degree of photodamage does not always correlate linearly with the extent of structural facial aging, particularly with regard to tissue laxity and volume loss of deeper facial compartments. This discrepancy highlights the limitations of the Glogau scale in describing global facial aging and supports the need for a three-dimensional clinical assessment integrating cutaneous, volumetric, and structural parameters during treatment planning.

Because of the descriptive case-series design, limited sample size, absence of a control group, and short follow-up, these findings should be considered preliminary. Further prospective studies with larger samples, control groups, longer follow-up, and standardized quantitative outcome measures are needed to evaluate the effectiveness, reproducibility, and duration of the observed improvements.

## 3. Discussion

The present preliminary case series describes short-term clinical and instrumental changes observed in three patients following a multimodal facial-rejuvenation pathway based on the PATH protocol. The treatment pathway combined chemical peeling, HA–succinate filler injection, intradermal biorevitalization, and homecare. Because several active interventions were administered within the same treatment sequence and no control or comparator group was included, the observed changes cannot be attributed to the PATH protocol as a whole or to any individual component.

From a clinical perspective, the three patients showed proportional changes in facial volume distribution, preservation of natural facial expression, and no visible overcorrection at the final follow-up. Instrumental assessments using OBSERV 520^®^, Antera 3D PRO^®^, and QuantifiCare LifeViz^®^ Infinity Pro documented changes in texture, brightness, surface regularity, and soft-tissue distribution. However, these findings should be interpreted exclusively as descriptive observations. In particular, improvements in skin texture and quality may have been influenced not only by the injectable treatments but also by chemical peeling and the homecare regimen, which included an active topical retinoid with an independent effect on skin appearance [47]. Therefore, the present case series does not allow conclusions regarding the efficacy of the complete PATH protocol or the relative contribution of its individual components.

These observations should be interpreted within current models of skin aging, in which fibroblast senescence, extracellular matrix degradation, oxidative stress, and impaired cellular metabolism contribute to progressive loss of dermal quality and function [44]. Since facial aging involves simultaneous changes in the epidermis, dermis, fat compartments, ligaments, muscles, and skeletal structures, a multimodal treatment strategy has a biological and anatomical rationale. Nevertheless, the present findings do not demonstrate that a combined approach is more effective than isolated treatments.

### 3.1. Biological and Clinical Rationale of the Combined Protocol

The PATH protocol is based on a multilayered treatment rationale [19]. Chemical peeling is intended to promote epidermal renewal and improve superficial texture and chromatic uniformity [48], whereas HA–succinate filler is administered in deeper planes for structural support and volume restoration [17,34,35,36]. Intradermal biorevitalization is intended to support hydration and visible skin quality [50], while homecare represents an additional active component of the treatment pathway, particularly because the regimen included a topical retinoid [47].

These intended effects provide the rationale for combining the individual procedures but should not be interpreted as demonstrated effects of the complete PATH protocol. Because chemical peeling, filler injection, biorevitalization, and active homecare were administered sequentially or concurrently, the contribution of each intervention cannot be isolated. Similarly, it is not possible to determine whether the observed changes resulted from one treatment, from several independent effects, or from additive or synergistic interactions among the different components.

However, these observations were descriptive and subjective and cannot establish that the combined strategy reduces the need for compensatory volumetric correction or provides superior outcomes compared with individual treatments.

### 3.2. Interpretation of Succinate and Biostimulatory Components

In the present case series, HA–succinate filler and polynucleotide-based biorevitalization were administered as components of a multimodal treatment pathway. Because no comparator filler without succinate was used and no treatment component was evaluated independently, the specific clinical contribution of succinate or polynucleotides cannot be distinguished from the volumetric and hydrating effects of HA or from the effects of the other interventions included in the treatment pathway.

The biological mechanisms proposed for succinate and polynucleotides, including their potential effects on cellular metabolism, fibroblast responses, tissue repair, and the dermal microenvironment, are discussed in the introductory literature review [43,44,45,52,53,59]. Similarly, the distinct biological rationale of structural biostimulatory materials such as calcium hydroxylapatite and poly-L-lactic acid, which have been associated with progressive tissue remodeling and neocollagenesis, is considered as background information rather than as a finding of the present case series [60,61,62].

No histological, biochemical, or molecular analyses were performed in the three patients. Therefore, fibroblast activation, extracellular matrix remodeling, collagen synthesis, or metabolic biostimulation cannot be directly demonstrated on the basis of the present findings. Accordingly, the observed clinical and instrumental changes should not be interpreted as evidence of any specific biological mechanism, nor do the present data support conclusions regarding the superiority or preferential indication of HA–succinate formulations compared with conventional HA fillers or other biostimulatory materials.

### 3.3. Clinical Implications, Limitations, and Future Perspectives

The combined use of peeling, filler, biorevitalization, and homecare reflects a multimodal approach to facial aging. However, the present findings should not be interpreted as demonstrating that simultaneous or sequential treatment of different tissue levels provides greater efficacy than individual interventions.

A particularly important limitation is the concurrent use of multiple active treatments. Chemical peeling, HA–succinate filler, intradermal biorevitalization, photoprotection, and topical homecare were all part of the therapeutic pathway. In particular, the topical retinoid represents an independent active intervention that may itself influence skin texture and quality [47]. Consequently, it is not possible to determine which component, or combination of components, was responsible for the changes observed at final follow-up. The specific clinical contribution of succinate likewise cannot be distinguished from the volumetric and hydrating effects of the HA-based filler.

The other principal limitations are the retrospective case-series design, the extremely small sample size, the absence of a control or comparator group, and the short follow-up period. The retrospective selection of cases with available clinical and instrumental documentation also introduces a potential risk of selection bias. Furthermore, some clinical outcomes, including facial harmony and overall skin quality, were based on subjective assessments and therefore require cautious interpretation.

No serious adverse events were reported in the three patients during the approximately 60-day observation period. However, this finding should not be interpreted as evidence of a favorable safety profile. The sample size and follow-up duration are insufficient to assess uncommon, delayed, or product-specific adverse events. For the same reasons, the observed clinical and instrumental changes cannot be considered evidence of efficacy.

Future studies should include larger patient cohorts, longer follow-up, standardized photographic and instrumental protocols, independent and preferably blinded assessments, validated patient-reported outcome measures, and appropriate control groups. Controlled comparisons with conventional HA fillers, non-succinate HA formulations, individual components of the multimodal pathway, and structural biostimulatory materials will be necessary to determine the specific contribution of each intervention and to evaluate whether the combined approach provides benefits beyond those attributable to its individual components.

## 4. Conclusions

This retrospective descriptive three-patient case series reports the short-term clinical and instrumental findings observed after a multimodal facial rejuvenation pathway combining chemical peeling, HA–succinate filler injection, intradermal biorevitalization, and homecare according to the PATH framework.

At approximately 60 days, the three cases showed descriptive changes in facial appearance, skin texture, chromatic uniformity, surface regularity, and soft-tissue distribution. These observations were documented by standardized photography and complementary instrumental assessments using OBSERV 520^®^, Antera 3D PRO^®^, and QuantifiCare LifeViz^®^ Infinity Pro. No serious adverse events were documented during the available follow-up period.

Because of the retrospective case-series design, the inclusion of only three patients, the absence of a control group, the simultaneous use of multiple active interventions, and the short follow-up, no conclusions regarding efficacy, safety, or the specific contribution of individual treatment components can be drawn. The present findings should therefore be considered descriptive and hypothesis-generating. Larger prospective controlled studies with longer follow-up are required to evaluate the clinical effectiveness, safety, reproducibility, and durability of this multimodal approach.

## Figures and Tables

**Table 1:** Products used in the PATH treatment framework.

Product	Product Type and Manufacturer	Declared Composition
Mesofiller^®^ Nexha Volume	Cross-linked hyaluronic acid filler; Mesoestetic Pharma Group, Barcelona, Spain	Sodium hyaluronate, 2.5% (25 mg/mL; MW 1.5–4.0 MDa), in a cross-linked hyaluronic acid gel; lidocaine hydrochloride, 3 mg/mL (0.3%). Other ingredients reported in the IFU: water for injection, succinic acid, sodium chloride, sodium hydroxide, and 1,4-butanediol di-glycidyl ether (BDDE).
Mesopeel^®^ MD Azelan RX	Superficial chemical peel; mesoestetic^®^, Barcelona, Spain	Azelaic acid, 18%; salicylic acid, 15%; lactic acid, 5%; ethanol; caffeic acid; tranexamic acid; glycyrrhetinic acid; water; shikimic acid; glycerin; arginine; retinal; xanthophylls.
mesohyal™ Organic Silicon	Intradermal product; Mesoestetic Pharma Group, Barcelona, Spain	Free hyaluronic acid (sodium hyaluronate), 2.5 mg/mL (12.5 mg/5 mL vial); organic silicon, 2.7 mg/5 mL, present as monomethylsilanetriol. Other components include sodium chloride, hydroxyproline, aspartic acid, salicylic acid, methylparahydroxybenzoate, propylparahy-droxybenzoate, and water for injection q.s. to 5 mL.
mesohyal™ X-DNA	Intradermal product; Mesoestetic Pharma Group, Barcelona, Spain	Free hyaluronic acid (sodium hyaluronate), 2.5 mg/mL (7.5 mg/3 mL vial); highly polymerized sodium deoxyribonucleotide (concentration not disclosed by the manufacturer). Other components reported in the IFU: sodium chloride, disodium phosphate, sodium edetate, and water for injection q.s. to 3 mL
Anti-Stress Mask	Post-procedural mask; mesoestetic^®^	Aqua, Propanediol, Glycerin, Caprylic/Capric Triglyceride, PEG-7 Glyceryl Cocoate, Tridecyl Stearate, Aloe Barbadensis Leaf Juice Powder, Rhodosorus Marinus Extract, Chamomilla Recutita Extract, Rosmarinus Officinalis Leaf Extract, Ascorbyl Palmitate, Ascorbic Acid, Alteromonas Ferment Extract, Biosaccharide Gum-2, Calendula Officinalis Flower Extract, Tocopherol, Lactobacillus Ferment, Crocus Sativus Flower Extract, Tocopheryl Acetate, Tridecyl Trimellitate, PEG/PPG-14/4 Dimethicone, Saccharide Isomerate, Acrylates/C10-30 Alkyl Acrylate Crosspolymer, Acrylamide/Sodium Acryloyldime-thyltaurate Copolymer, Propylene Glycol, Isohexadecane, Dipentae-rythrityl Hexacaprylate/Hexacaprate, Menthyl Lactate, Sodium Hy-droxide, Disodium EDTA, Helianthus Annuus Seed Oil, Polysorbate 80, Maltodextrin, PEG-8, Sorbitan Oleate, Butylene Glycol, Citric Acid, Sodium Citrate, Ethylhexylglycerin, Phenoxyethanol.
Post-Procedure Gel Cream	Post-procedural topical product; mesoestetic^®^	Aqua, Dicaprylyl Carbonate, Cetearyl Alcohol, Caprylic/Capric Tri-glyceride, Cyclopentasiloxane, Polyacrylate-13, Glyceryl Stearate, PEG-100 Stearate, Diisopropyl Adipate, Sodium DNA, Ethylhexyl-glycerin, Dimethylmethoxy Chromanol, Glyceryl Caprylate, Butylene Glycol, Sodium Hyaluronate, Ulmus davidiana Root Extract, Tocopherol, Phenoxyethanol, Propanediol, Cetearyl Glucoside, Poly-isobutene, Disodium EDTA, Lecithin, Acrylic Acid/Acrylamidomethyl Propane Sulfonic Acid Copolymer, Polysorbate 20, Sorbitan Isostearate, Xanthan Gum, Parfum.
Age Element Firming Concentrate	Homecare topical product; mesoestetic^®^	Aqua, Sodium Polyacrylate Starch, Glycerin, Dipropylene Glycol, Shikimic Acid, Avena Sativa Kernel Extract, Calanthe Discolor Extract, Canola Oil, Carnosine, Caesalpinia Spinosa Fruit Extract, Helianthus Annuus Seed Oil, Limnanthes Alba Seed Oil, Argania Spinosa Kernel Oil, Kappaphycus Alvarezii Extract, Gleditsia Triacanthos Seed Extract, Gluconolactone, Caprylyl Glycol, Calcium Gluconate, Acetyl Tetrapeptide-2, Ethylhexylglycerin, Polyglyceryl-10 Stearate, Ferulic Acid, Maslinic Acid, Isoquercetin, Propanediol, Microcrystalline Cellulose, Xanthan Gum, Cellulose Gum, Polyacrylate Crosspolymer-6, Disodium EDTA, Sodium Benzoate, Phenoxyethanol.
Skinretin 0.3%	Topical retinoid product; mesoestetic^®^	Pure retinol (Retinol), 0.3%; bakuchiol, 0.7%. Complete INCI: Aqua, Glycerin, Lactococcus Ferment Lysate, Isononyl Isononanoate, Propanediol, Sodium Acrylates Copolymer, Centella Asiatica Leaf Extract, Bakuchiol, Ectoin, Retinol, Bisabolol, Helianthus Annuus Seed Oil, Lactic Acid, Sunflower Seed Oil Glycerides, Serine, Rosmarinus Officinalis Leaf Extract, Undecane, Ethylhexylglycerin, DATEM, Simethicone, Allantoin, Urea, Sorbitol, Sodium Lactate, Tridecane, Pentylene Glycol, Tocopheryl Acetate, Citric Acid, Lecithin, Polysorbate 20, Sclerotium Gum, Xanthan Gum, Polysilicone-11, Sodium Chloride, Disodium EDTA, BHT, BHA, Sodium Benzoate, Phenoxyethanol.
Mesoprotech^®^ Water Veil SPF 50+	Broad-spectrum sunscreen; mesoestetic^®^	Broad-spectrum UVA/UVB photoprotection; SPF 50+ (very high UVB protection); very high UVA protection (PPD rating: very high). Complete INCI: Aqua, Ethylhexyl Methoxycinnamate, Octocrylene, Diisopropyl Adipate, Alcohol Denat., Bis-Ethylhexyloxyphenol Methoxyphenyl Triazine, Butyl Methoxydibenzoylmethane, Silica, Methylene Bis-Benzotriazolyl Tetramethylbutylphenol, C15-19 Alkane, Isostearyl Alcohol, Butylene Glycol Cocoate, Propylene Glycol, Capryloyl Glycerin/Sebacic Acid Copolymer, Titanium Dioxide, Polyacrylate Crosspolymer-6, Dicaprylyl Carbonate, Bis-Ethylhexyl Hydroxydimethoxy Benzylmalonate, Helianthus Annuus Seed Oil, Lupinus Albus Seed Extract, Rosmarinus Officinalis Leaf Extract, Plankton Extract, Sodium Hyaluronate, Argania Spinosa Kernel Oil, Persea Gratissima Oil, Lecithin, Hydroxytyrosol, Dimethicone, Ethylhexylglycerin, Sucrose, Lactic Acid, Tocopherol, Tocophersolan, Methoxy PEG-150 Butyl Methacrylate/Methacryloyloxyethoxy Methylcoumarin Copolymer, Glycerin, Propanediol, Decyl Glucoside, Ethylcellulose, Xanthan Gum, Tetrasodium Glutamate Diacetate, Phenoxyethanol, Parfum.

**Table 2:** Individual treatment schedule and administered doses.

Patient	Session and Date	Chemical Peeling	HA–Succinate Filler	Intradermal Biorevitalization	Homecare and Follow-Up
Patient 1	Baseline: (16 October 2025/day 0)	(2 mL); two layers; 5 min; neutralized with 4–5 sprays of Post-Peel Neutralizing Spray and subsequently removed with a water-moistened sponge.	(2 mL total); areas: (zygomatic region, mandibular angle, jawline, nasolabial folds, and labiomandibular folds); planes: (supraperiosteal and deep dermal); technique: (supraperiosteal needle bolus injections in the zygomatic region and mandibular angle, and deep-dermal linear retrograde threading with a cannula in the zygomatic region, jawline, nasolabial folds, and labiomandibular folds).	Organic Silicon (2.5 mL); X-DNA (3 mL); (mixed)	Post-Procedure Gel Cream, approximately 1–2 mL twice daily until resolution of erythema/skin reactivity; Age Element Firming Concentrate, 3–4 drops twice daily after resolution of skin reactivity and throughout the treatment period; Skinretin 0.3%, thin layer every other evening, initiated 5 days after each peeling session after complete resolution of irritation and discontinued 5 days before the subsequent peeling session; Mesoprotech^®^ Water Veil SPF 50+, approximately 2 mg/cm^2^ every morning, reapplied every 2 h during prolonged outdoor exposure. Direct sun exposure was avoided throughout the treatment period; intense physical activity, saunas, and facial massage for 48 h after each procedure; potentially irritating topical products for 5 days or until complete resolution of erythema/irritation, whichever occurred later.
Patient 1	Session 2: (31 October 2025/day 15)	(2 mL); two layers; 5 min; neutralized with 4–5 sprays of Post-Peel Neutralizing Spray and subsequently removed with a water-moistened sponge.	None	Organic Silicon (2.5 mL); X-DNA (3 mL); (mixed)	—
Patient 1	Session 3: (15 November 2025/day 30)	(2 mL); two layers; 5 min; neutralized with 4–5 sprays of Post-Peel Neutralizing Spray and subsequently removed with a water-moistened sponge.	None	Organic Silicon (2.5 mL); X-DNA (3 mL); (mixed)	—
Patient 1	Session 4: (1 December 2025/day 46)	(2 mL); two layers; 5 min; neutralized with 4–5 sprays of Post-Peel Neutralizing Spray and subsequently removed with a water-moistened sponge.	None	Organic Silicon (2.5 mL); X-DNA (3 mL); (mixed)	—
Patient 1	Final assessment: (15 December 2025/day 60)	—	—	—	Clinical, photographic, instrumental, and GAIS assessment
Patient 2	Baseline: (16 October 2025/day 0)	(2 mL); two layers; 5 min; neutralized with 4–5 sprays of Post-Peel Neutralizing Spray and subsequently removed with a water-moistened sponge.	(2 mL total); areas: (lower facial third, cheek region, mandibular region, nasolabial folds, and labiomandibular folds); planes: (supraperiosteal and deep dermal); technique: (supraperiosteal needle bolus injections in the mandibular region and deep-dermal linear retrograde threading with a cannula in the cheek and mandibular regions, nasolabial folds, and labiomandibular folds).	Organic Silicon (2.5 mL); X-DNA (3 mL); (mixed)	Post-Procedure Gel Cream, approximately 1–2 mL twice daily until resolution of erythema/skin reactivity; Age Element Firming Concentrate, 3–4 drops twice daily after resolution of skin reactivity and throughout the treatment period; Skinretin 0.3%, thin layer every other evening, initiated 5 days after each peeling session after complete resolution of irritation and discontinued 5 days before the subsequent peeling session; Mesoprotech^®^ Water Veil SPF 50+, approximately 2 mg/cm^2^ every morning, reapplied every 2 h during prolonged outdoor exposure. Direct sun exposure was avoided throughout the treatment period; intense physical activity, saunas, and facial massage for 48 h after each procedure; potentially irritating topical products for 5 days or until complete resolution of erythema/irritation, whichever occurred later.
Patient 2	Session 2: (31 October 2025/day 15)	(2 mL); two layers; 5 min; neutralized with 4–5 sprays of Post-Peel Neutralizing Spray and subsequently removed with a water-moistened sponge.	None	Organic Silicon (2.5 mL); X-DNA (3 mL); (mixed)	—
Patient 2	Session 3: (15 November 2025/day 30)	(2 mL); two layers; 5 min; neutralized with 4–5 sprays of Post-Peel Neutralizing Spray and subsequently removed with a water-moistened sponge.	None	Organic Silicon (2.5 mL); X-DNA (3 mL); (mixed)	—
Patient 2	Session 4: (1 December 2025/day 46)	(2 mL); two layers; 5 min; neutralized with 4–5 sprays of Post-Peel Neutralizing Spray and subsequently removed with a water-moistened sponge.	None	Organic Silicon (2.5 mL); X-DNA (3 mL); (mixed)	—
Patient 2	Final assessment: (15 December 2025/day 60)	—	—	—	Clinical, photographic, instrumental, and GAIS assessment
Patient 3	Baseline: (16 October 2025/d/day 0)	(2 mL); two layers; 5 min; neutralized with 4–5 sprays of Post-Peel Neutralizing Spray and subsequently removed with a water-moistened sponge.	(4 mL total); areas: (zygomatic region, mandibular region, chin, nasolabial folds, and labiomandibular folds); planes: (supraperiosteal and deep dermal); technique: (supraperiosteal needle bolus injections in the zygomatic region, mandibular region, and chin, and deep-dermal linear retrograde threading with a cannula in the zygomatic and mandibular regions, nasolabial folds, and labiomandibular folds).	Organic Silicon (2.5 mL); X-DNA (3 mL); (mixed)	Post-Procedure Gel Cream, approximately 1–2 mL twice daily until resolution of erythema/skin reactivity; Age Element Firming Concentrate, 3–4 drops twice daily after resolution of skin reactivity and throughout the treatment period; Skinretin 0.3%, thin layer every other evening, initiated 5 days after each peeling session after complete resolution of irritation and discontinued 5 days before the subsequent peeling session; Mesoprotech^®^ Water Veil SPF 50+, approximately 2 mg/cm^2^ every morning, reapplied every 2 h during prolonged outdoor exposure. Direct sun exposure was avoided throughout the treatment period; intense physical activity, saunas, and facial massage for 48 h after each procedure; potentially irritating topical products for 5 days or until complete resolution of erythema/irritation, whichever occurred later.
Patient 3	Session 2: (31 October 2025/day 15)	(2 mL); two layers; 5 min; neutralized with 4–5 sprays of Post-Peel Neutralizing Spray and subsequently removed with a water-moistened sponge.	None	Organic Silicon (2.5 mL); X-DNA (3 mL); (mixed)	—
Patient 3	Session 3: (15 November 2025/day 30)	(2 mL); two layers; 5 min; neutralized with 4–5 sprays of Post-Peel Neutralizing Spray and subsequently removed with a water-moistened sponge.	None	Organic Silicon (2.5 mL); X-DNA (3 mL); (mixed)	—
Patient 3	Session 4: (1 December 2025/day 46)	(2 mL); two layers; 5 min; neutralized with 4–5 sprays of Post-Peel Neutralizing Spray and subsequently removed with a water-moistened sponge.	None	Organic Silicon (2.5 mL); X-DNA (3 mL); (mixed)	—
Patient 3	Final assessment: (15 December 2025/day 60)	—	—	—	Clinical, photographic, instrumental, and GAIS assessment

**Table 3:** Clinical Characteristics, Treatment Details, and Follow-Up of the Three Included Cases.

Case	Sex	Age	Clinical Presentation	Treated Area	Filler Volume	Assessments	Follow-Up
1	Female	52	Moderate facial aging with both cutaneous and structural involvement, reduced malar convexity, mild lower-face laxity, diffuse dyschromia, and irregular skin texture	Middle and lower thirds of the face	2 mL	OBSERV 520^®^, Antera 3D PRO^®^, QuantifiCare LifeViz^®^ Infinity Pro	60 days
2	Female	52	Moderate facial aging with predominant perioral and genian involvement, superficial soft-tissue depletion, perioral static wrinkles, dyschromia, and texture irregularity	Perioral/genian region, middle and lower thirds	2 mL	OBSERV 520^®^, Antera 3D PRO^®^, QuantifiCare LifeViz^®^ Infinity Pro	60 days
3	Female	58	Moderate-to-advanced facial aging with greater structural involvement, lower-face laxity, midface volume loss, and reduced definition of anatomical transitions	Middle and lower thirds of the face	4 mL	OBSERV 520^®^, Antera 3D PRO^®^, QuantifiCare LifeViz^®^ Infinity Pro	60 days

**Table 4:** Treatment-Related Local Reactions Observed During the PATH Treatment Pathway and Their Duration.

Treatment	Observed Local Reaction(s)	Approximate Duration	Management
Chemical peeling	Mild transient erythema	<24 h	Spontaneous resolution; routine post-procedural skincare
HA–succinate filler injection	Mild edema and occasional mild ecchymosis at the injection sites	Edema: 24–48 h; ecchymosis: up to 72 h	Spontaneous resolution; no additional medical treatment
Intradermal biorevitalization	Mild localized erythema and edema	24–48 h	Spontaneous resolution; no additional medical treatment
Homecare regimen	No treatment-related adverse reactions documented	—	—

**Table 5:** Summary of clinical and photographic outcomes at T60.

Case	Main Baseline Features	Main Clinical Changes at T60	Overcorrection	Facial Expression
1	Reduced malar convexity, mild lower-face laxity, diffuse dyschromia, irregular texture	Improved facial harmony, better midface continuity, increased brightness and skin uniformity	No	Preserved
2	Perioral soft-tissue depletion, static wrinkles, genian dyschromia and texture irregularity	Improved perioral volume continuity, reduced wrinkle visibility, greater chromatic uniformity	No	Preserved
3	Lower-face laxity, midface volume loss, reduced definition of anatomical transitions	Improved volumetric balance, greater continuity of facial contours, more harmonious appearance	No	Preserved

**Table 6:** Summary of Antera 3D PRO^®^ findings at T60.

Case	Main Improved Parameters	Interpretation
1	Fine lines, texture, wrinkles/furrows, pigmentation uniformity	Improved microrelief, compactness, and skin-tone homogeneity
2	Fine lines, texture/roughness, wrinkles, pores, pigmentation	Improved surface regularity, reduced discontinuities, greater chromatic uniformity
3	Malar wrinkles, fine lines, texture, indentation/elevation volumes	Improved skin surface continuity and malar microrelief regularity

**Table 7:** Summary of QuantifiCare LifeViz^®^ Infinity Pro findings at T60.

Case	Vector Pattern	Volumetric Findings	Interpretation
1	Mild, homogeneous, cranio-lateral vectors in the midface	No measurable quantitative volumetric parameters available	Harmonious soft-tissue redistribution
2	Cranio-lateral vectors in the midface, without discordant focal vectors	Indicative increases of approximately 1.9–2.9 mm in malar and mandibular regions	Improved support and projection
3	Cranio-lateral vectors, mainly in the midface	Bilateral increases of approximately 2.02 mm and 1.87 mm in zygomatic-malar regions	Improved bilateral midface support and volumetric balance

## Data Availability

The original contributions presented in this study are included in the article/Supplementary Materials. Further inquiries can be directed to the corresponding author.

## Supplementary Materials

The following supporting information can be downloaded at https://www.mdpi.com/article/10.3390/reports9030282/s1: Figure S1: Complete standardized clinical photographic documentation of Case 1 before treatment and at T60 after the PATH protocol; Figure S2: Complete OBSERV 520^®^ qualitative assessment of Case 1 before treatment and after the PATH protocol; Figure S3: Complete Antera 3D PRO^®^ quantitative assessment of Case 1 before treatment and at T60 after the PATH protocol; Figure S4: Complete QuantifiCare LifeViz^®^ Infinity Pro. three-dimensional assessment of Case 1 before treatment and at T60 after the PATH protocol; Figure S5: Complete standardized clinical photographic documentation of Case 2 before treatment and at T60 after the PATH protocol; Figure S6: Complete OBSERV 520^®^ qualitative assessment of Case 2 before treatment and at T60 after the PATH protocol; Figure S7: Complete Antera 3D PRO^®^ quantitative assessment of Case 2 before treatment and at T60 after the PATH protocol; Figure S8: Complete QuantifiCare LifeViz^®^ Infinity Pro three-dimensional assessment of Case 2 before treatment and at T60 after the PATH protocol; Figure S9: Complete standardized clinical photographic documentation of Case 3 before treatment and at T60 after the PATH protocol; Figure S10: Complete OBSERV 520^®^ qualitative assessment of Case 3 before treatment and at T60 after the PATH protocol; Figure S11: Complete Antera 3D PRO^®^ quantitative assessment of Case 3 before treatment and at T60 after the PATH protocol; Figure S12: Complete QuantifiCare LifeViz^®^ Infinity Pro. Three-dimensional assessment of Case 3 before treatment and at T60 after the PATH protocol.

## Abbreviations

The following abbreviations are used in this manuscript:

3D	Three-dimensional
A2A	Adenosine A2A receptor
BDDE	1,4-butanediol diglycidyl ether
CD44	Cluster of differentiation 44
COL1A1	Collagen type I alpha 1 chain
FBN1	Fibrillin-1
GAIS	Global Aesthetic Improvement Scale
HA	Hyaluronic acid
IL	Interleukin
MMP	Matrix metalloproteinase
PATH	Profundity, Action, Timing, and Home care
RHAMM	Receptor for hyaluronan-mediated motility
Smad	Small mothers against decapentaplegic
SPF	Sun protection factor
T0	Baseline
T60	Sixty-day follow-up
TGF-β	Transforming growth factor beta
TLR	Toll-like receptor
UV	Ultraviolet
