# Supplementary material for: The PATH Protocol for Integrated Facial Rejuvenation: A Preliminary Three-Patient Case Report Series and Narrative Review of the Literature
Source: Reports (MDPI). 2026 Aug 25;9(3):282. doi: 10.3390/reports9030282 (PMC13611034; doi:10.3390/reports9030282)
Supplement: Supplementary file 1 [file reports-09-00282-s001.zip › reports-4456329-supplementary.pdf]

Supplementary Materials

Supplementary Figure S1. Complete Clinical Photographic Documentation of Case 1

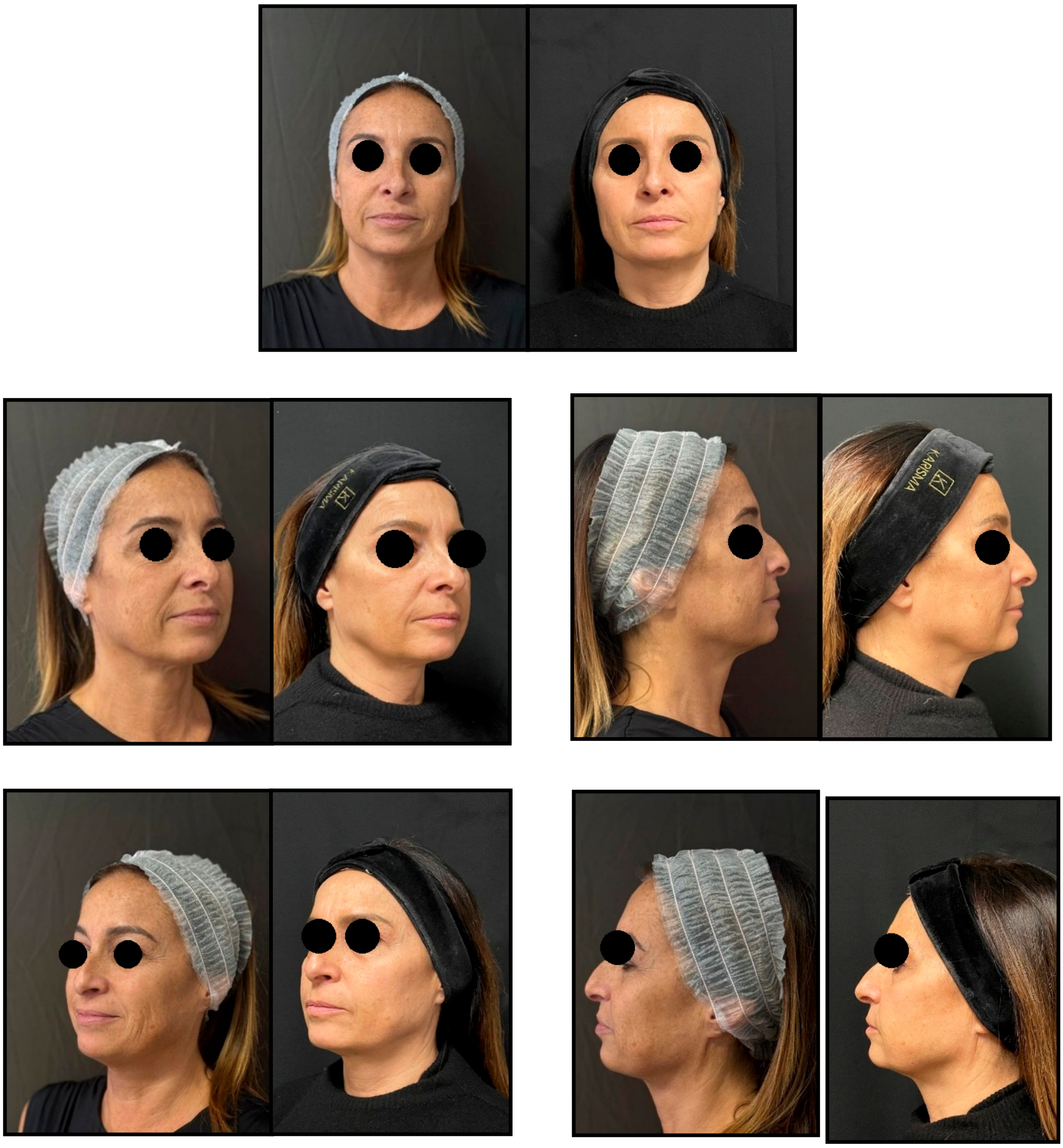

Figure S1. Complete standardized clinical photographic documentation of Case 1 before treatment and at T60 after the PATH protocol.

Pre-treatment images are shown in the left column and post-treatment T60 images in the right column. The panel includes frontal, right and left three-quarter, and right and left lateral views. At T60, the images show improved facial harmony, greater continuity of the midface volume, increased skin brightness and uniformity, and preservation of natural facial expression, without evidence of overcorrection.

Supplementary Figure S2. OBSERV 520® Qualitative Assessment

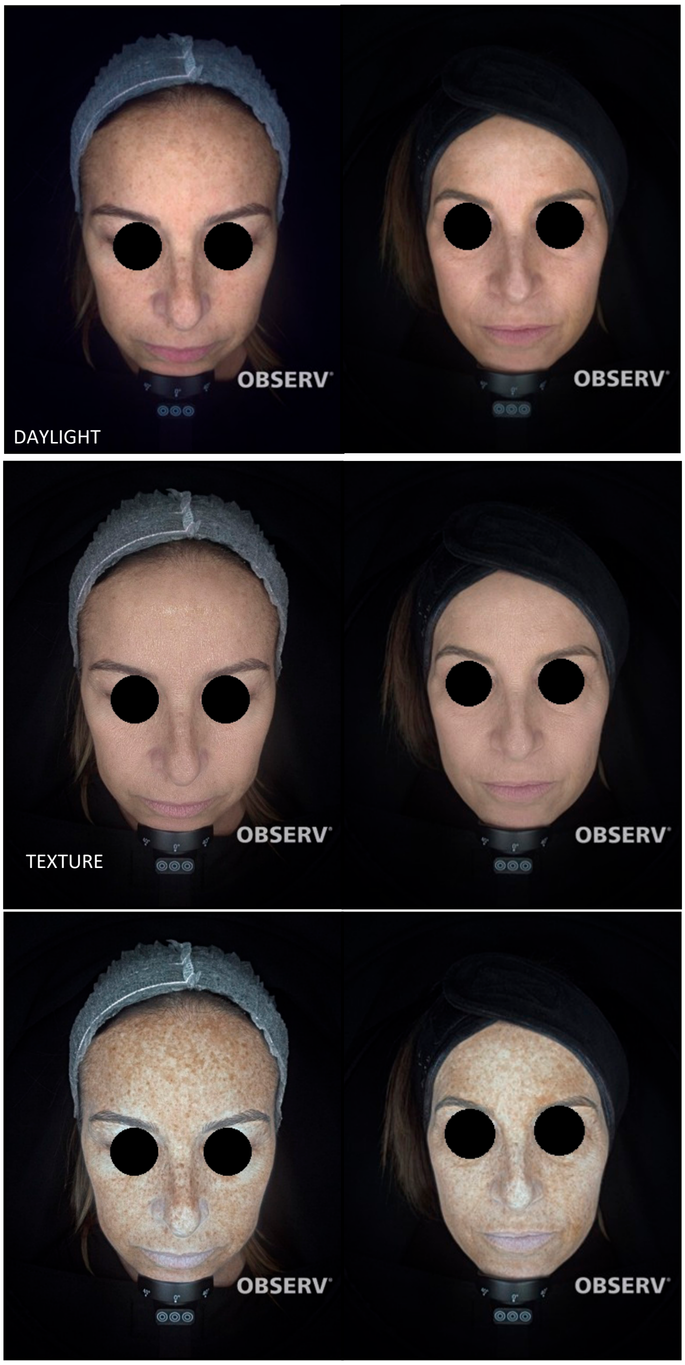

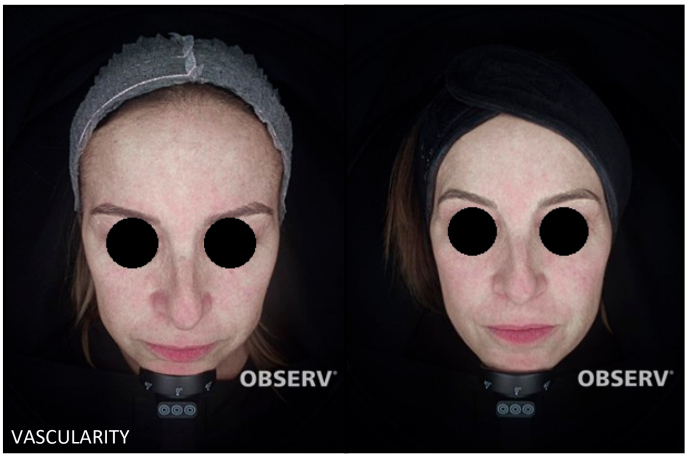
OBSERV
OBSERV
VASCULARITY

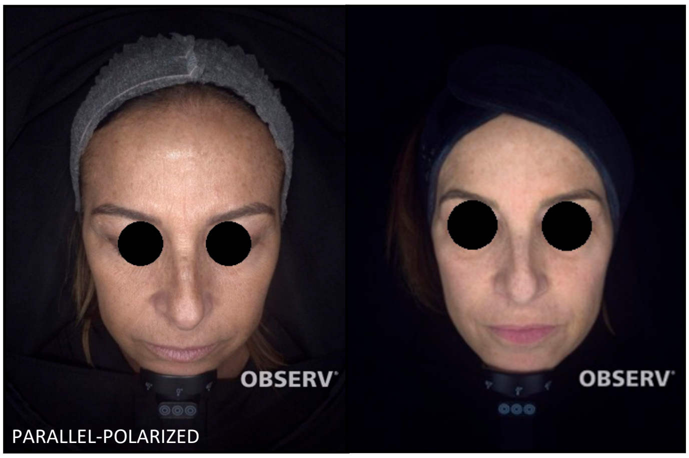
OBSERV
OBSERV
PARALLEL-POLARIZED

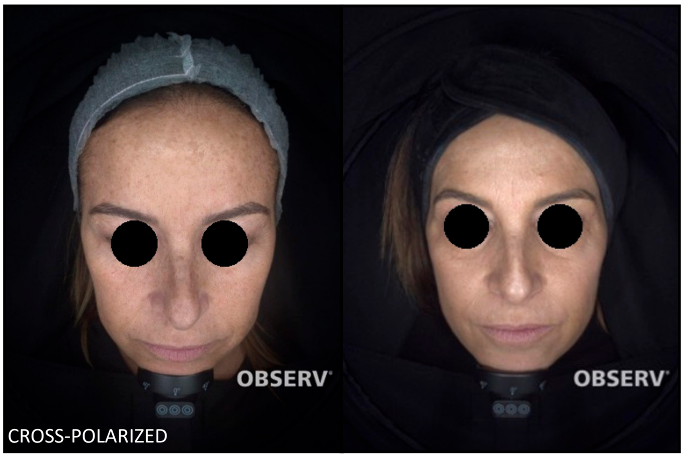
OBSERV
OBSERV
CROSS-POLARIZED

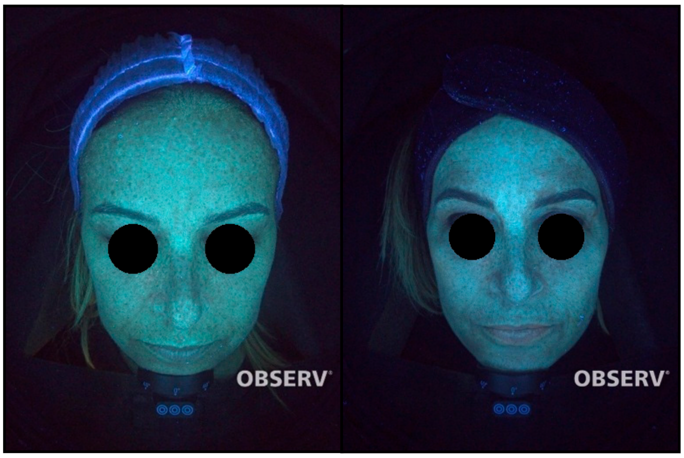
OBSERV
OBSERV

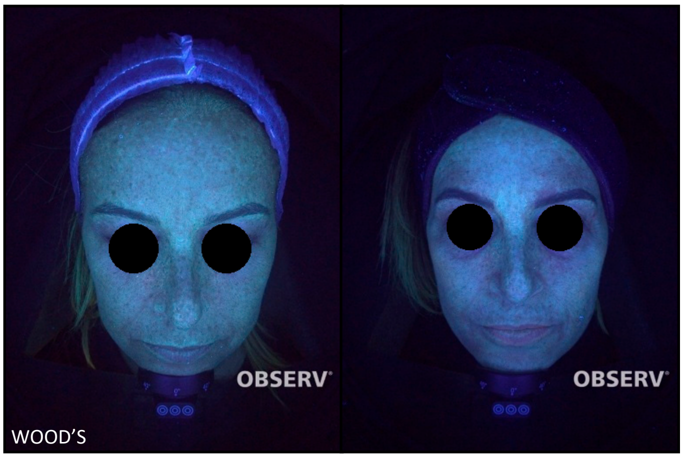

Figure S2. Complete OBSERV 520® qualitative assessment of Case 1 before treatment and after the PATH protocol.
Pre-treatment images are shown in the left column and post-treatment images in the right column. The panel includes Daylight, Texture, Pigmentation, Vascularity, Parallel-Polarized, Cross-Polarized, True UV, and Wood's light modes. The images document qualitative changes in skin texture, chromatic uniformity, superficial pigmentation, and vascular-related features after treatment.

Supplementary Figure S3. Antera 3D PRO® Quantitative Assessment

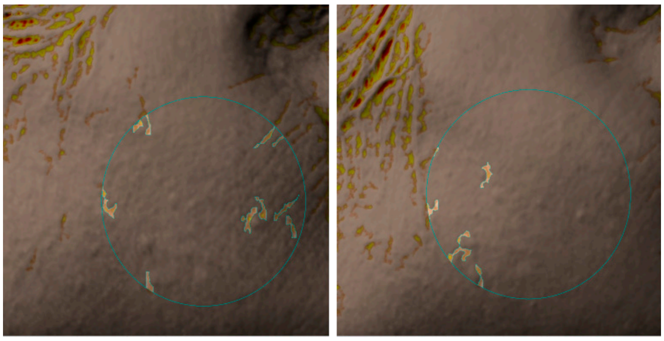

Wrinkles

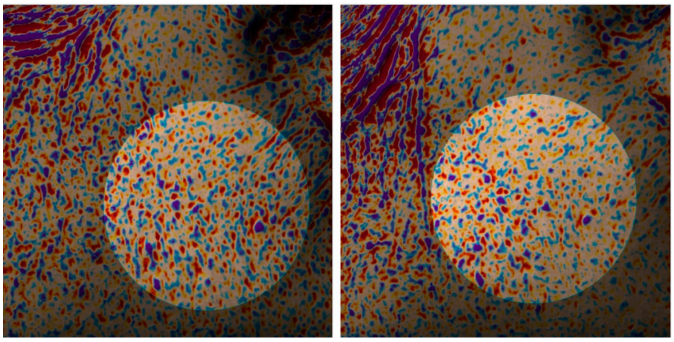

Skin texture/Roughness

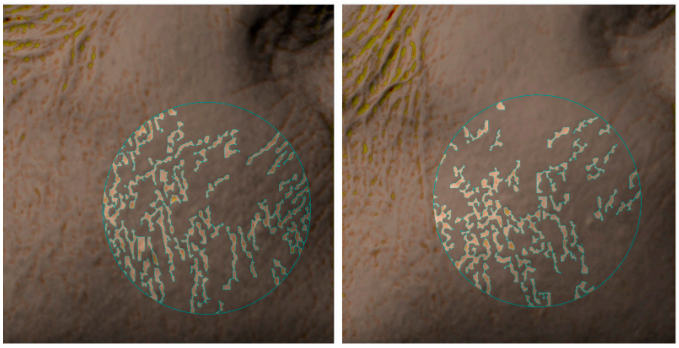

Free Lines – 3D Surface Map

Figure S3. Complete Antera 3D PRO® quantitative assessment of Case 1 before treatment and at T60 after the PATH protocol.
Selected three-dimensional maps show changes in wrinkles, skin texture/roughness, fine lines, and 3D surface morphology. Pre-treatment images are shown in the left column and post-treatment T60 images in the right column. The findings are consistent with improved skin surface regularity, smoother cutaneous texture, and greater uniformity of the cutaneous microrelief in the treated areas.

.

Supplementary Figure S4. QuantifiCare LifeViz® Infinity Pro. Three-Dimensional Assessment

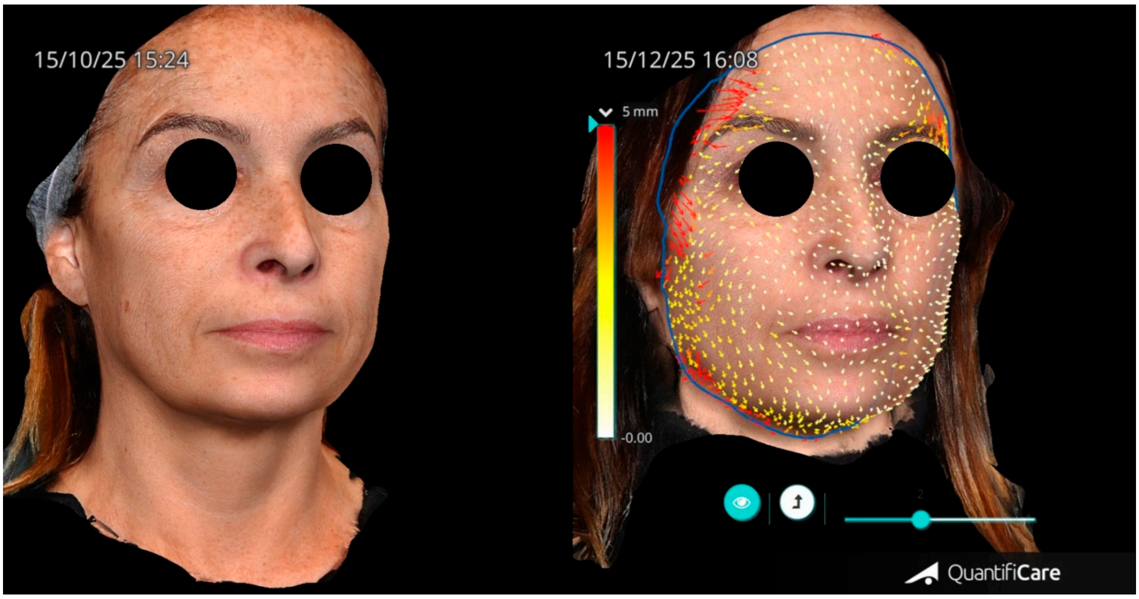

Figure S4. Complete QuantifiCare LifeViz® Infinity Pro. three-dimensional assessment of Case 1 before treatment and at T60 after the PATH protocol.

Pre-treatment images are shown in the left column and post-treatment T60 images in the right column. The analysis documents three-dimensional soft-tissue changes and superficial tissue displacement after treatment, supporting a more harmonious redistribution of facial volumes without clinically relevant asymmetry or overcorrection.

Supplementary Figure S5. Complete Clinical Photographic Documentation of Case 2

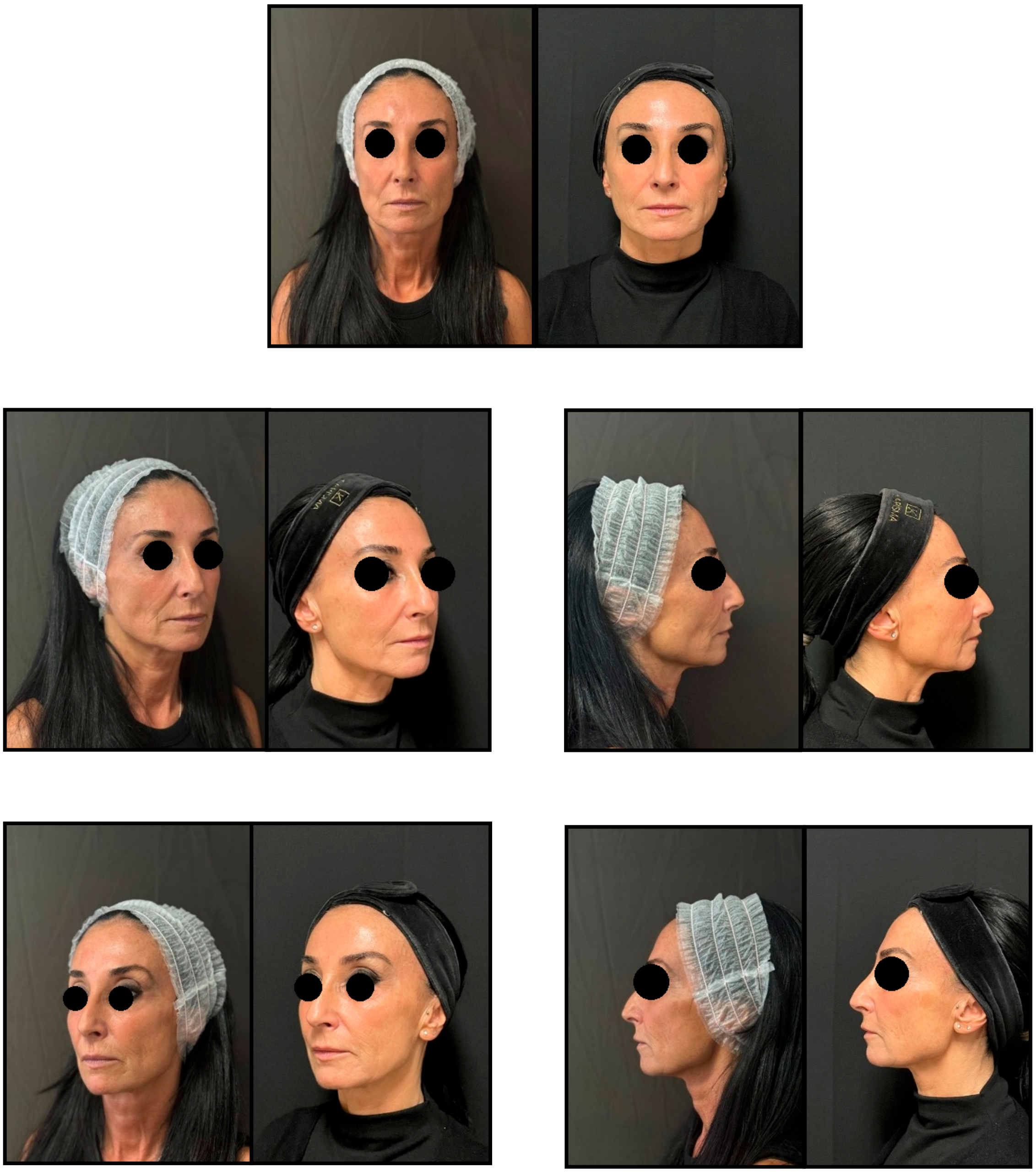

Figure S5. Complete standardized clinical photographic documentation of Case 2 before treatment and at T60 after the PATH protocol.

Pre-treatment images are shown in the left column and post-treatment T60 images in the right column. The panel includes frontal, right and left three-quarter, and right and left lateral views. At T60, the images show improved perioral and genian volume continuity, greater chromatic uniformity, smoother facial transitions, and preservation of natural facial expression, without evidence of overcorrection.

Supplementary Figure S6. OBSERV 520® Qualitative Assessment of Case 2

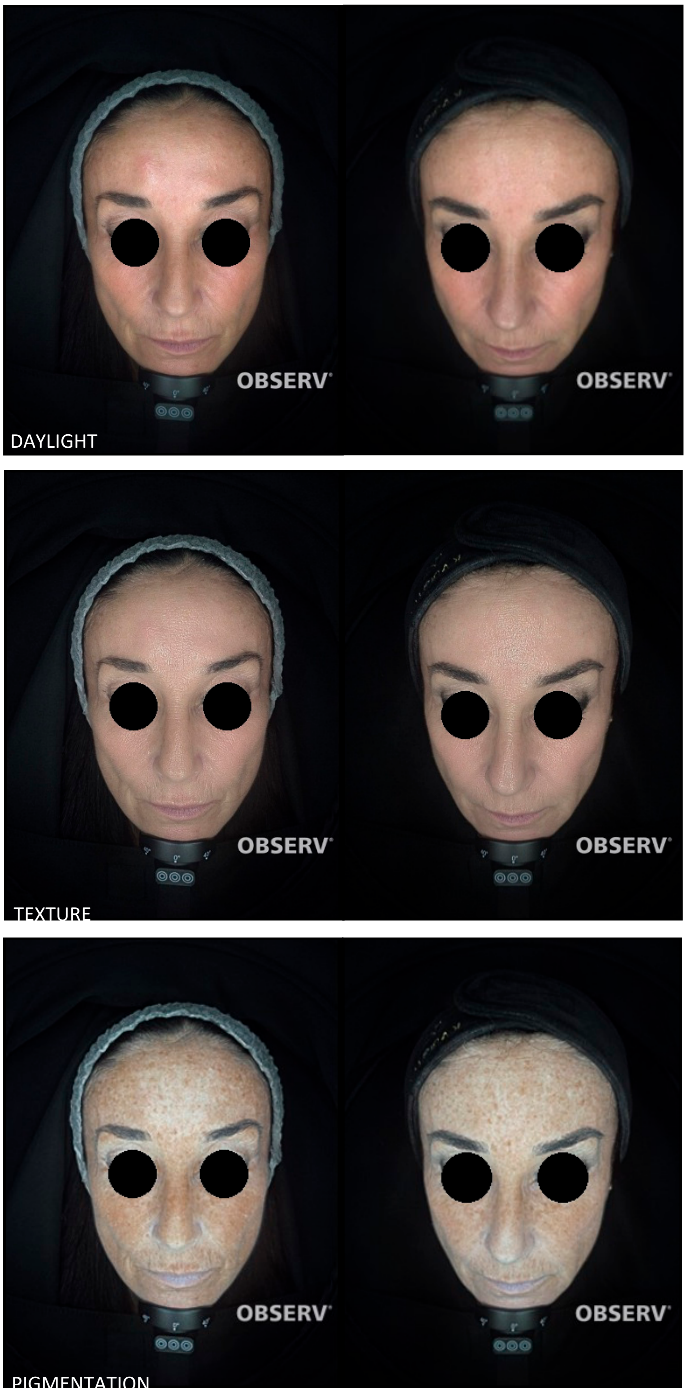

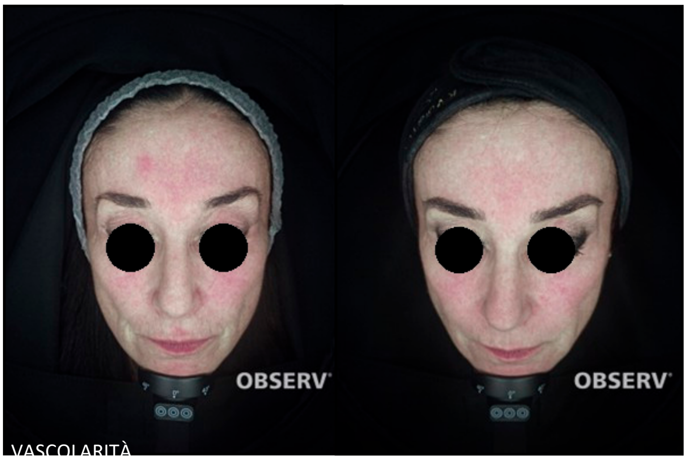

VASCOLARITÀ

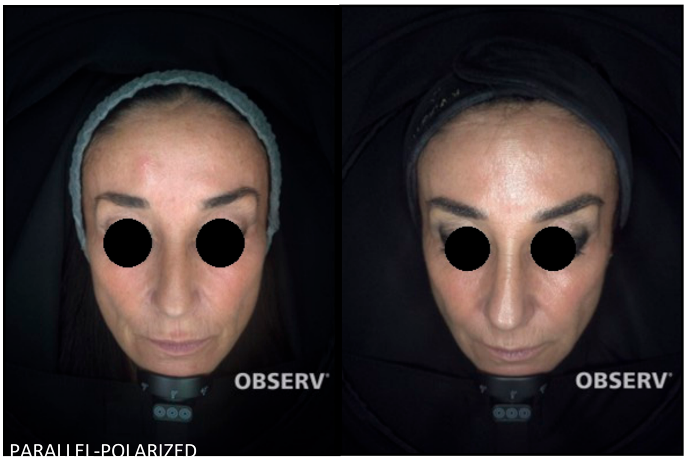

PARALLEL-POLARIZED

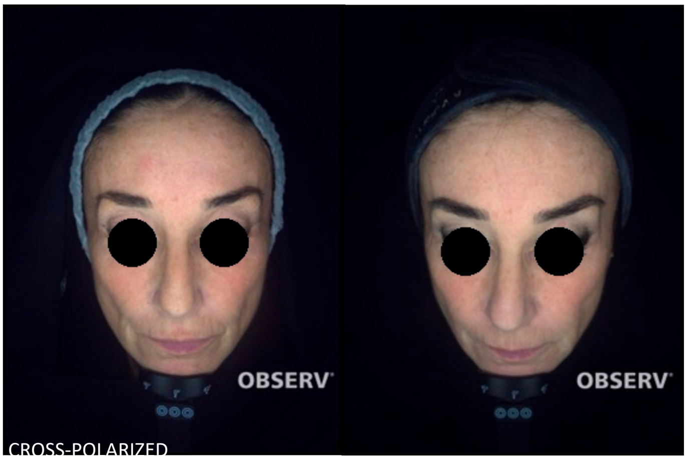

CROSS-POLARIZED

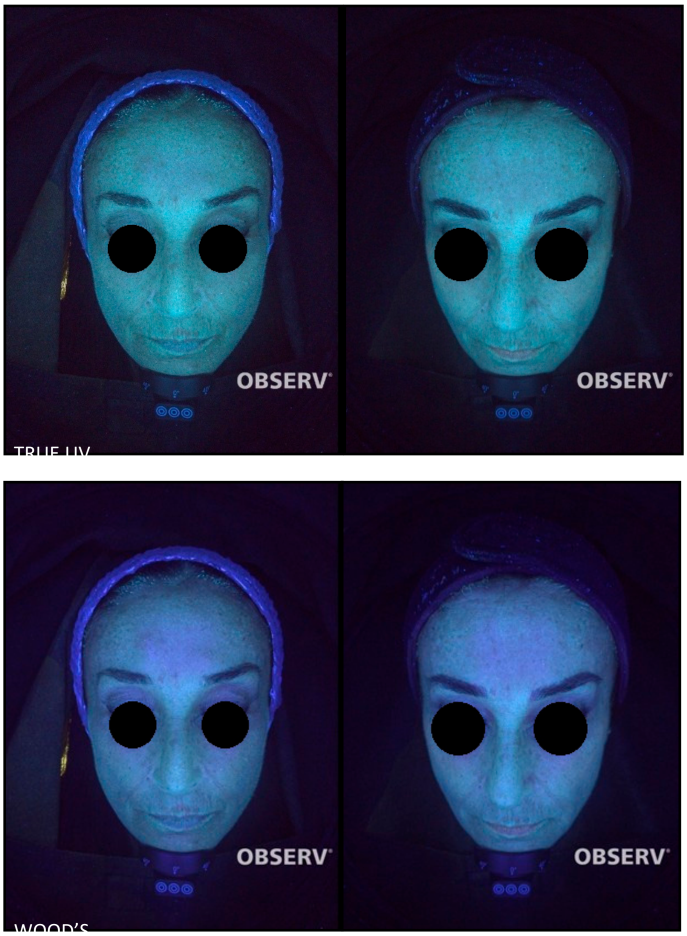

Figure S6. Complete OBSERV 520® qualitative assessment of Case 2 before treatment and at T60 after the PATH protocol.

Pre-treatment images are shown in the left column and post-treatment T60 images in the right column. The panel includes Daylight, Texture, Pigmentation, Vascularity, Parallel-Polarized, Cross-Polarized, True UV, and Wood’s light modes. The images document qualitative changes in skin texture, chromatic uniformity, superficial pigmentation, and vascular-related features after treatment.

Supplementary Figure S7. Antera 3D PRO® Quantitative Assessment of Case 2

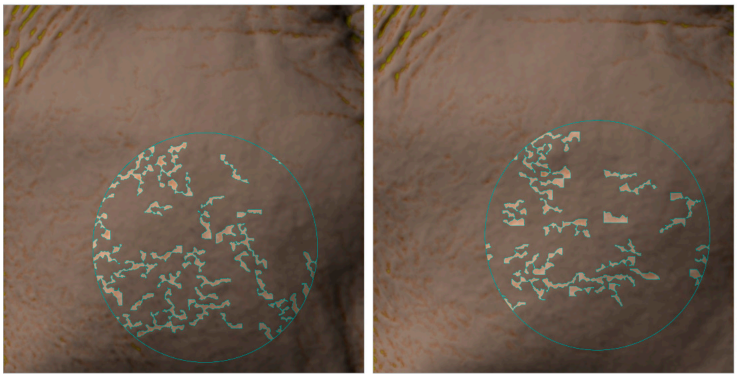

Fine Lines

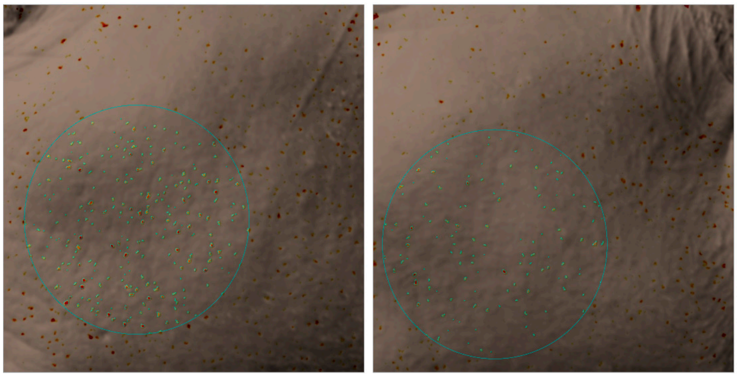

Pores

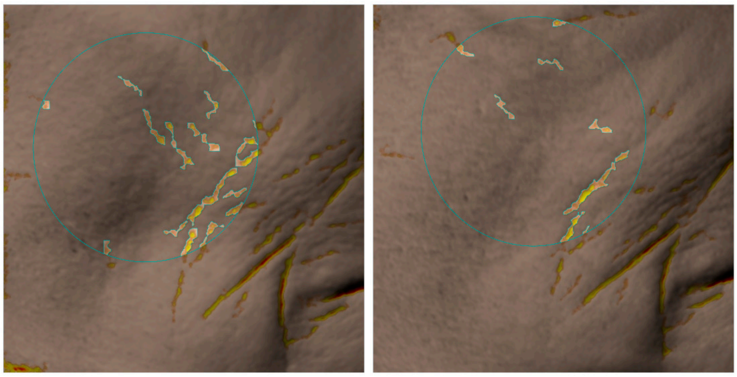

Wrinkles

Figure S7. Complete Antera 3D PRO® quantitative assessment of Case 2 before treatment and at T60 after the PATH protocol.

Selected three-dimensional maps show changes in fine lines, pores, wrinkles, and overall skin surface morphology. Pre-treatment images are shown in the left column and post-treatment T60 images in the right column. The findings are consistent with improved skin surface regularity and qualitative improvement of the cutaneous texture in the treated areas.

Supplementary Figure S8. QuantifiCare LifeViz® Infinity Pro. Three-Dimensional Assessment of Case 2

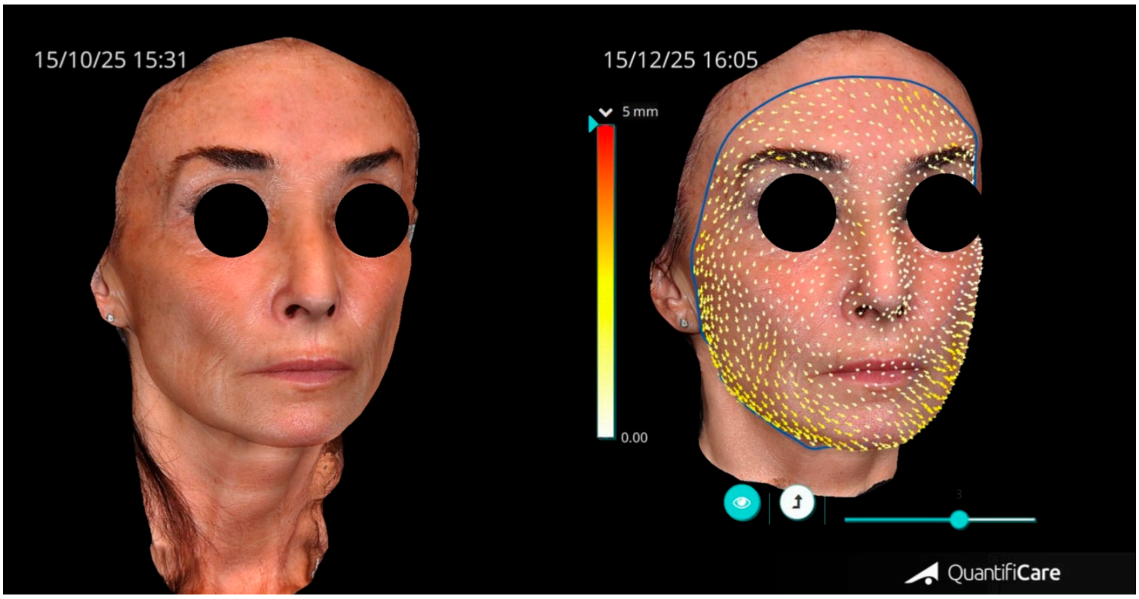

A

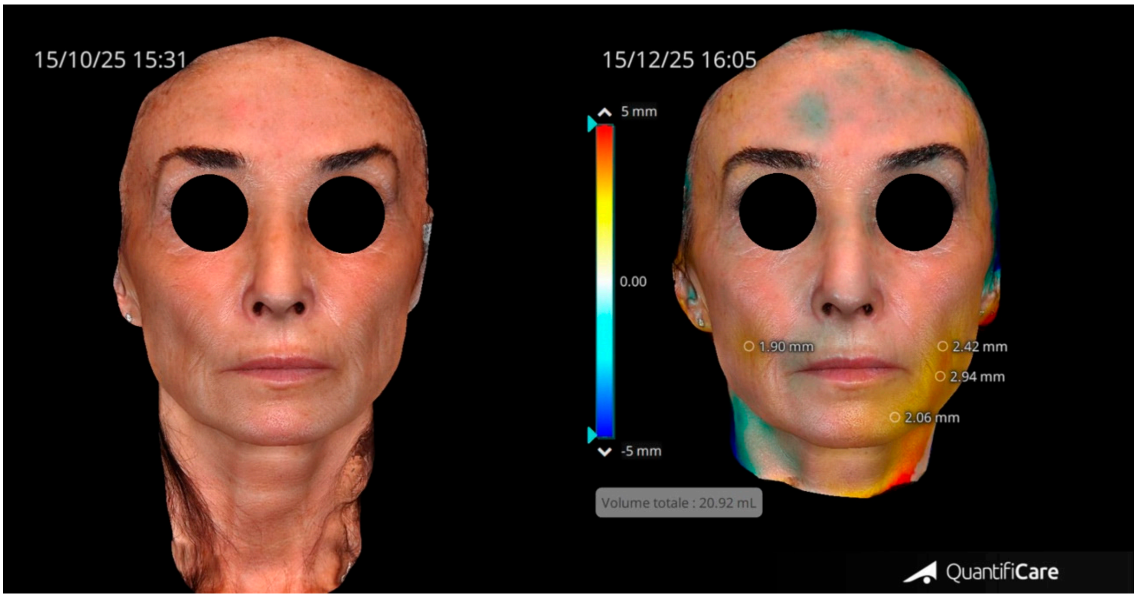

B

Figure S8. Complete QuantifiCare LifeViz® Infinity Pro. three-dimensional assessment of Case 2 before treatment and at T60 after the PATH protocol.

Pre-treatment images are shown in the left column and post-treatment T60 images in the right column. **(A)** Soft-tissue displacement vector map. **(B)** Superficial volumetric variation map. The analysis documents three-dimensional tissue repositioning and volumetric changes after treatment, supporting improved facial support and a more harmonious redistribution of facial volumes.

Supplementary Figure S9. Complete Clinical Photographic Documentation of Case 3

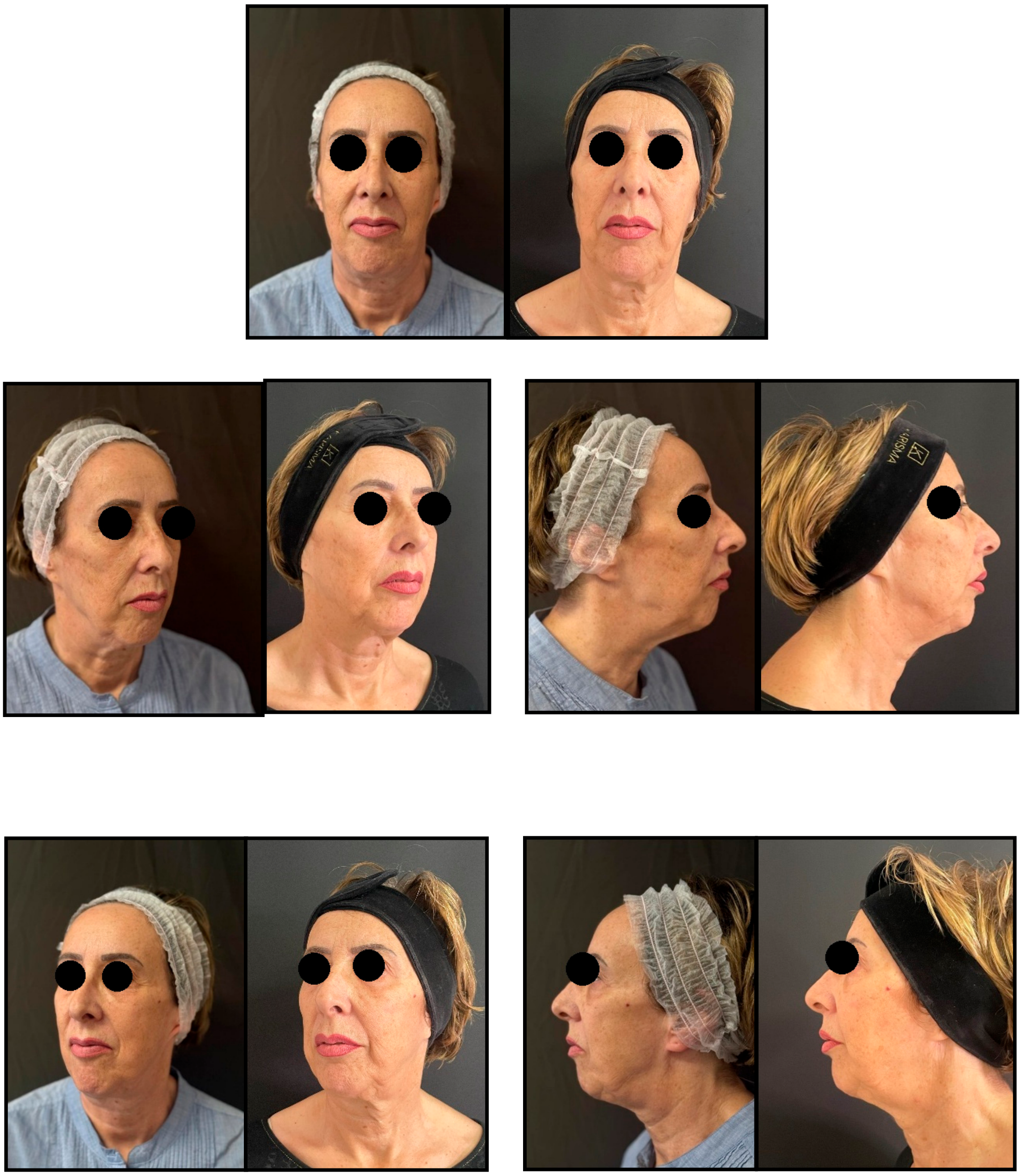

Figure S9. Complete standardized clinical photographic documentation of Case 3 before treatment and at T60 after the PATH protocol.

Pre-treatment images are shown in the left column and post-treatment T60 images in the right column. The panel includes frontal, right and left three-quarter, and right and left lateral views. At T60, the images show improved volumetric balance, greater continuity of facial contours,

better definition of the middle and lower thirds, and preservation of natural facial expression, without evidence of overcorrection.

Supplementary Figure S10. OBSERV 520® Qualitative Assessment of Case 3

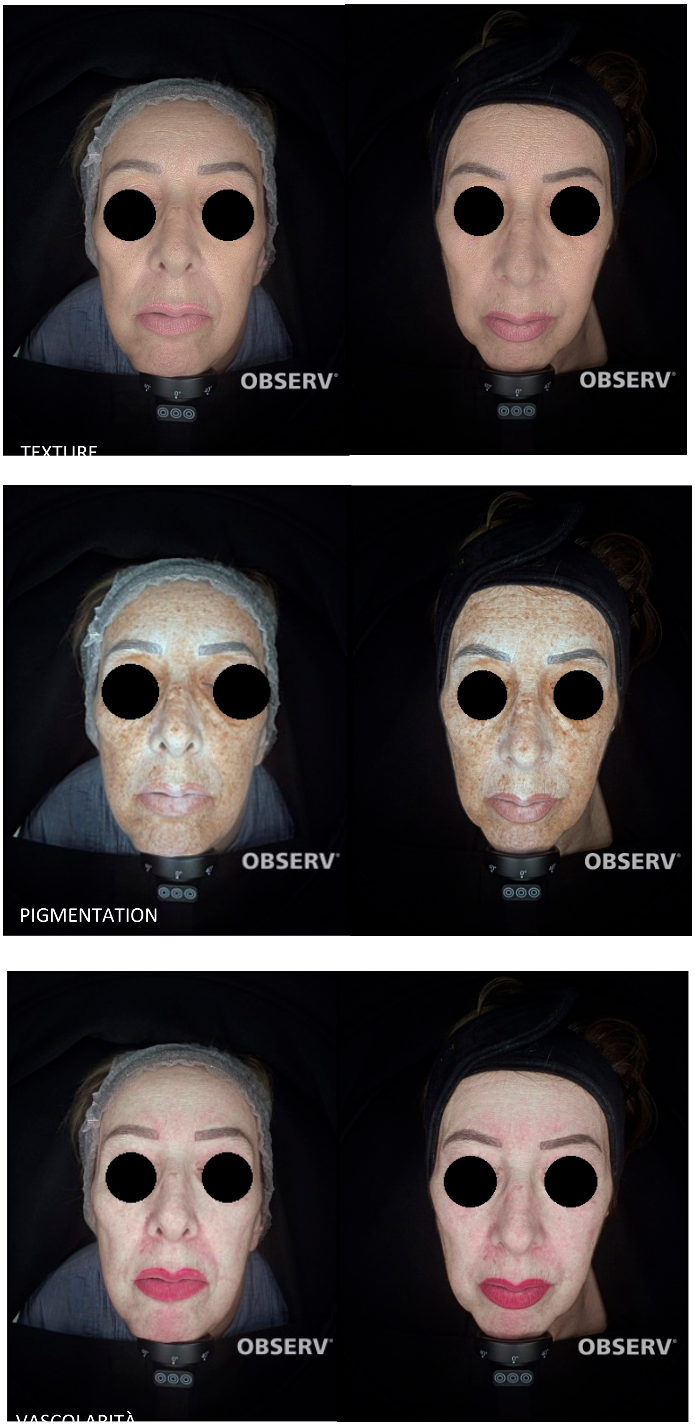

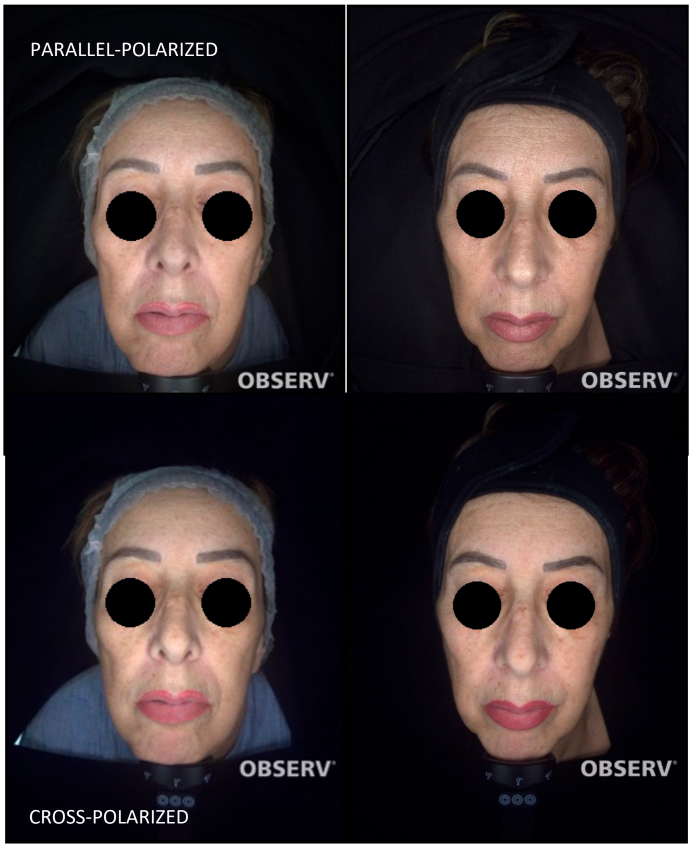
PARALLEL-POLARIZED
OBSERV
OBSERV
OBSERV
OBSERV
CROSS-POLARIZED

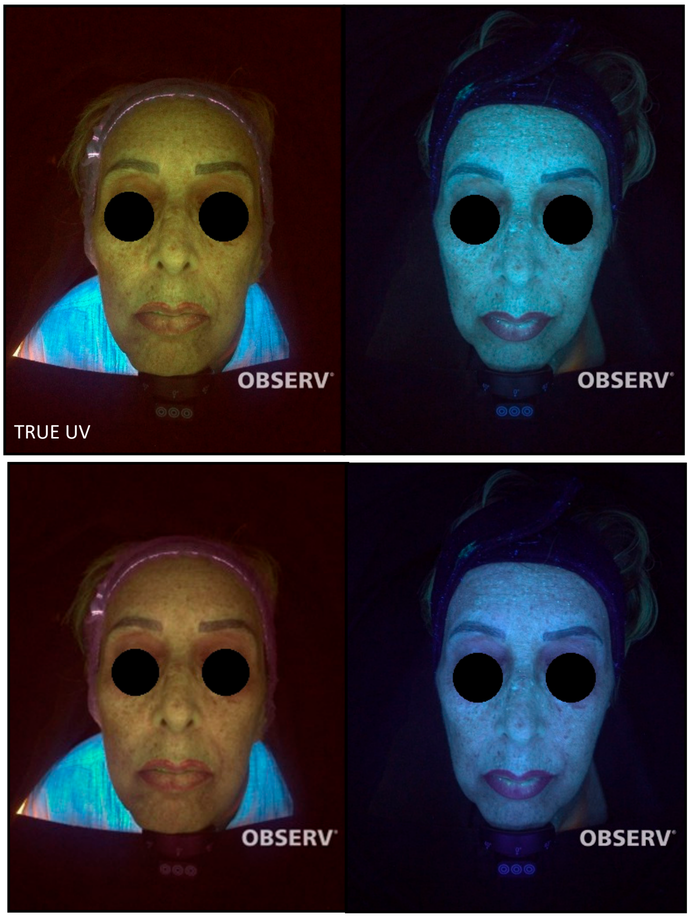
OBSERV
OBSERV
TRUE UV
OBSERV
OBSERV

Figure S10. Complete OBSERV 520® qualitative assessment of Case 3 before treatment and at T60 after the PATH protocol.

Pre-treatment images are shown in the left column and post-treatment T60 images in the right column. The panel includes Daylight, Texture, Pigmentation, Vascularity, Parallel-Polarized, Cross-Polarized, True UV, and Wood's light modes. The images document qualitative changes in skin texture, chromatic uniformity, superficial pigmentation, and vascular-related features after treatment.

Supplementary Figure S11. Antera 3D PRO® Quantitative Assessment of Case 3

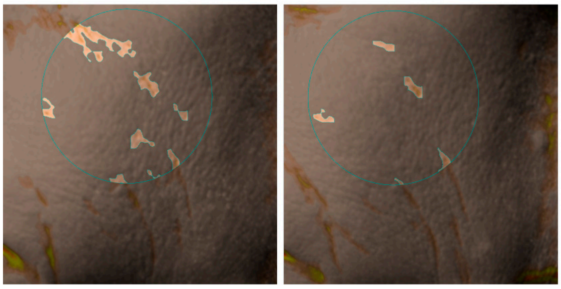

Furrows

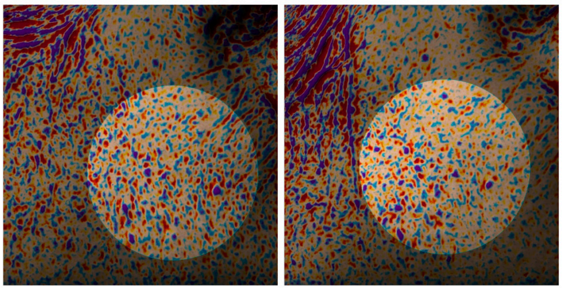

SKIN TEXTURE / ROUGHNESS

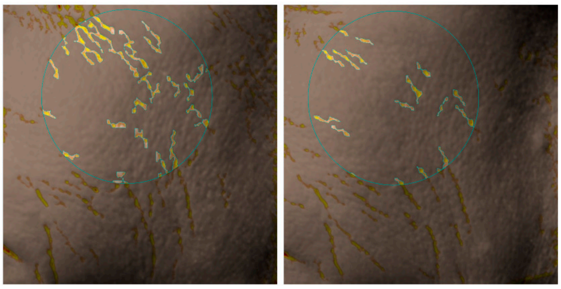

WRINKLES

Figure S11. Complete Antera 3D PRO® quantitative assessment of Case 3 before treatment and at T60 after the PATH protocol.

Selected three-dimensional maps show changes in furrows, skin texture/roughness, wrinkles, and overall skin surface morphology. Pre-treatment images are shown in the left column and post-treatment T60 images in the right column. The findings are consistent with improved skin

surface regularity, smoother cutaneous texture, and greater uniformity of the cutaneous microrelief in the treated areas.

Supplementary Figure S12. QuantifiCare LifeViz® Infinity Pro. Three-Dimensional Assessment of Case 3

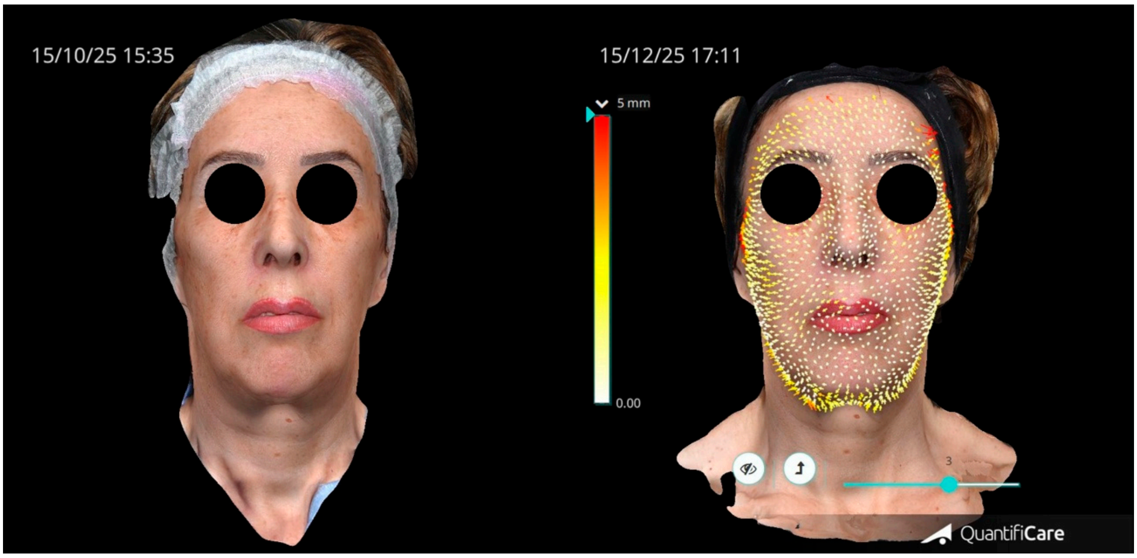

A

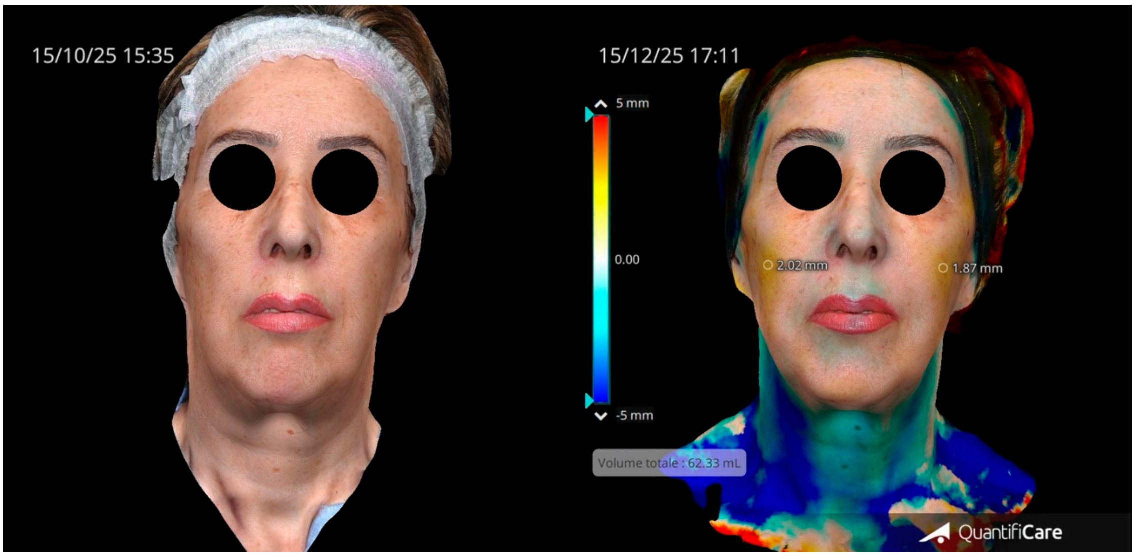

B

Figure S12. Complete QuantifiCare LifeViz® Infinity Pro. Three-dimensional assessment of Case 3 before treatment and at T60 after the PATH protocol.

Pre-treatment images are shown in the left column and post-treatment T60 images in the right column. (A) Soft-tissue displacement vector map. (B) Superficial volumetric variation map. The analysis documents three-dimensional soft-tissue displacement and superficial volumetric changes after treatment, supporting improved bilateral midface support, better volumetric balance, and a more harmonious redistribution of facial volumes.
